# Attachment Goes to Court: Child Protection and Custody Issues

**DOI:** 10.1080/14616734.2020.1840762

**Published:** 2021-01-11

**Authors:** Tommie Forslund, Pehr Granqvist, Marinus H. van IJzendoorn, Avi Sagi-Schwartz, Danya Glaser, Miriam Steele, Mårten Hammarlund, Carlo Schuengel, Marian J. Bakermans-Kranenburg, Howard Steele, Phillip R. Shaver, Ulrike Lux, John Simmonds, Deborah Jacobvitz, Ashley M. Groh, Kristin Bernard, Chantal Cyr, Nancy L. Hazen, Sarah Foster, Elia Psouni, Philip A. Cowan, Carolyn Pape Cowan, Anne Rifkin-Graboi, David Wilkins, Blaise Pierrehumbert, George M. Tarabulsy, Rodrigo A. Carcamo, Zhengyan Wang, Xi Liang, Maria Kázmierczak, Paulina Pawlicka, Lilian Ayiro, Tamara Chansa, Francis Sichimba, Haatembo Mooya, Loyola McLean, Manuela Verissimo, Sonia Gojman-de-Millán, Marlene M. Moretti, Fabien Bacro, Mikko J. Peltola, Megan Galbally, Kiyomi Kondo-Ikemura, Kazuko Y. Behrens, Stephen Scott, Andrés Fresno Rodriguez, Rosario Spencer, Germán Posada, Rosalinda Cassibba, Neus Barrantes-Vidal, Jesus Palacios, Lavinia Barone, Sheri Madigan, Karen Mason-Jones, Sophie Reijman, Femmie Juffer, R. Pasco Fearon, Annie Bernier, Dante Cicchetti, Glenn I. Roisman, Jude Cassidy, Heinz Kindler, Peter Zimmerman, Ruth Feldman, Gottfried Spangler, Charles H. Zeanah, Mary Dozier, Jay Belsky, Michael E. Lamb, Robbie Duschinsky

**Affiliations:** Department of Psychology, Stockholm University, Stockholm, Sweden, SUF Resource Center, Region Uppsala, Uppsala, Sweden; Department of Psychology, Stockholm University, Stockholm, Sweden; Department of Psychology, Education and Child Studies, Erasmus University Rotterdam, The Netherlands; School of Psychological Sciences and Center for the Study of Child Development, University of Haifa, Israel; Great Ormond Street Hospital, London, UK, and Department of Clinical, Educational and Health Psychology, University College London, UK; Psychology Department, The New School, New York, NY, USA; Department of Psychology, Stockholm University, Stockholm, Sweden; Faculty of Behavioural and Movement Sciences, Clinical Child and Family Studies, Vrije Universiteit Amsterdam, The Netherlands; Psychology Department, The New School, New York, NY, USA; University of California, Davis, CA, USA; Department Families and Family Policies, German Youth Institute, Munich, Germany; British Association for Adoption and Fostering at Coram (CoramBAAF); Department of Human Development and Family Sciences, University of Texas at Austin, TX, USA; Department of Psychology, University of Missouri-Columbia, Columbia, MO, USA; Department of Psychology, Stony Brook University, NY, USA; Département de Psychologie, Université du Québec à Montréal, Canada; Department of Human Development and Family Sciences, University of Texas at Austin, TX, USA; Department of Social Work, Education and Community Wellbeing, Northumbria University, Newcastle, UK; Department of Psychology, Lund University, Sweden; Department of Psychology, University of California, Berkeley, CA, USA; Singapore Institute for Clinical Sciences (SICS), Agency for Science and Technology (A*STAR), Singapore; School of Social Sciences, Cardiff University, Cardiff; University of Lausanne, Switzerland; School of Psychology, Laval University, Québec City, Canada; Department of Psychology, University of Magallanes, Chile; Research Centre for Child Development, Beijing Key Laboratory of Learning and Cognition, School of Psychology, Capital Normal University, Beijing, China; Institute of Psychology, Faculty of Social Sciences, University of Gdansk, Poland; Department of Educational Psychology, Maseno University, Kenya; Department of Psychology, University of Zambia, Lusaka, Zambia; Brain and Mind Centre, Faculty of Medicine and Health, The University of Sydney, Australia; William James Center for Research, ISPA – Instituto Universitário, Lisboa, Portugal; Research Center of the Seminario de Sociopsicoanálisis, Mexico City, Mexico; Department of Psychology, Simon Fraser University, Vancouver, Canada; Faculté de Psychologie, Université de Nantes, France; Human Information Processing Laboratory, Psychology, Faculty of Social Sciences, Tampere University, Finland; College of Science, Health, Education and Engineering, Murdoch University, Australia, School of Medicine, University of Notre Dame, Australia, King Edward Memorial Hospital, Australia; Department of Psychology, Teikyo University, Tokyo, Japan; Department of Social & Behavioral Sciences, State University of New York Polytechnic Institute, Utica, NY, USA; Institute of Psychiatry, Psychology and Neuroscience, Kings’s College London, UK; Department of Psychology, University of Talca, Chile; Department of Human Development and Family Studies, Purdue University, West Lafayette, IN, USA; Department of Educational Sciences, Psychology, Communication, University of Bari, Italy; Departament de Psicologia Clínica i de la Salut, Universitat Autònoma de Barcelona, Spain, Sant Pere Claver – Fundació Sanitària, Barcelona, Spain, Centre for Biomedical Research Network on Mental Health (CIBERSAM), Barcelona, Spain; Department of Developmental and Educational Psychology, University of Seville, Spain; Department of Brain and Behavioral Sciences, Lab of Attachment and Parenting - LAG, University of Pavia, Italy; Department of Psychology, University of Calgary, AB, Alberta Children’s Hospital Research Institute, AB, Canada; Center for Health & Community, University of California, San Francisco, CA, USA; Department of Public Health and Primary Care, University of Cambridge, UK; Centre for Child and Family Studies, Leiden University, The Netherlands; Research Department of Clinical, Educational and Health Psychology, University College London, London, UK; Département de Psychologie, Université de Montréal, Canada; Institute of Child Development and Department of Psychiatry, University of Minnesota, MN, USA; Institute of Child Development, University of Minnesota, Minneapolis, MN, USA; Department of Psychology, University of Maryland, College Park, MD, USA; German Youth Institute, Munich, Germany; Department of Psychology/Developmental Psychology, University of Wuppertal, Wuppertal, Germany; Baruch Ivcher School of Psychology, Interdisciplinary Center, Herzliya, Israel, & Child Study Center, Yale University, New Haven, CT, USA; Department of Psychology, Friedrich-Alexander-University of Erlangen-Nuremberg, Germany; Institute of Infant and Early Childhood Mental Health, Tulane University School of Medicine, New Orleans, LA, USA; Department of Psychological and Brain Sciences, University of Delaware, Newark, DE, USA; Department of Human Ecology, University of California, Davis, CA, USA; Department of Psychology, University of Cambridge, UK; Department of Public Health and Primary Care, University of Cambridge, UK

**Keywords:** attachment theory, best interests of the child, child custody, child protection, family court, consensus statement

## Abstract

Attachment theory and research are drawn upon in many applied settings, including family courts, but misunderstandings are widespread and sometimes result in misapplications. The aim of this consensus statement is, therefore, to enhance understanding, counter misinformation, and steer family-court utilisation of attachment theory in a supportive, evidence-based direction, especially with regard to child protection and child custody decision-making. The article is divided into two parts. In the first, we address problems related to the use of attachment theory and research in family courts, and discuss reasons for these problems. To this end, we examine family court applications of attachment theory in the current context of the best-interest-of-the-child standard, discuss misunderstandings regarding attachment theory, and identify factors that have hindered accurate implementation. In the second part, we provide recommendations for the application of attachment theory and research. To this end, we set out three attachment principles: the child’s need for familiar, non-abusive caregivers; the value of continuity of good-enough care; and the benefits of networks of attachment relationships. We also discuss the suitability of assessments of attachment quality and caregiving behaviour to inform family court decision-making. We conclude that assessments of caregiver behaviour should take center stage. Although there is dissensus among us regarding the use of assessments of attachment quality to inform child custody and child-protection decisions, such assessments are currently most suitable for targeting and directing supportive interventions. Finally, we provide directions to guide future interdisciplinary research collaboration.

Attachment theory and research have vast practical utility for those concerned with the well-being of children, caregiving, and family functioning. This has been evident since Bowlby’s early work on the adverse effects of major child-caregiver separations, which contributed to changes in hospital practice and allowed children greater accessibility to their parents when needed the most (Bowlby et al., 1952). Also, caregiver sensitivity has long been established as an important predictor of children’s attachment quality (Ainsworth et al., 1978; De Wolff & van IJzendoorn, 1997) across many cultures (Posada et al., 2016). Children’s attachment quality has in turn been repeatedly linked to the child’s development (Groh et al., 2017a; Vaughn et al., 2019). Finally, attachment theory and research have generated a number of evidence-based caregiving interventions with beneficial outcomes for children and their caregivers (Steele & Steele, 2017). Attachment theory and research have, consequently, become very influential and are currently put into practice in many applied settings, including family court^1^ assessment and decision-making (Alexius & Hollander, 2014; Crittenden & Baim, 2017). However, misinformation about attachment theory and research is widespread and sometimes results in misapplications with potentially serious negative consequences (for discussions, see Granqvist et al., 2017; Kelly & Lamb, 2000; Nielsen, 2014).

## Purpose and Aims

Our aim with this consensus statement is, therefore, to counter misinformation and help steer family court applications of attachment theory in a supportive, evidence-based direction on matters related to *child protection* and *custody decisions*. There are already various papers offering guidance for court practice based on attachment theory and research (e.g., Byrne et al., 2005; Smith et al., 2012a), but marginal opinions have sometimes been presented as reflecting a consensus position (e.g. Hacker & Halperin Kaddari, 2013; Schore & McIntosh, 2011). As academic scholars and practitioners, with long histories of studying and utilising attachment theory, our goal is to offer a measured consensus position based on the concerted body of attachment research. We consider both child protection and child custody because, despite important differences, there are sufficient similarities in terms of the basic principles at stake from an attachment perspective to permit joint consideration. The paper comprises two parts.

Part I, comprising three major headings, addresses problems related to the use of attachment theory and research in family courts.

(1) We examine family court applications of attachment theory *in the current context of the best-interest-of-the-child standard*.
(2) We discuss *central misunderstandings* regarding: (a) the nature of attachment, (b) the interaction among multiple attachment relationships, and (c) the implications of classifications of attachment quality.
(3) We identify *factors that have hindered accurate reception and utilisation* of attachment theory among family court practitioners, including the pressure for decision-making to appear evidence-based and the circulation of inaccurate accounts of attachment theory.

Part II, also comprising three major headings, provides recommendations for the application of attachment theory and research in the family courts.

(4) We advance three *fundamental principles* that court practitioners can use regarding attachment in individual cases: (a) the need for familiar, non-abusive caregivers; (b) the value of continuity of good-enough care; and (c) the benefits of networks of attachment relationships.
(5) We discuss *the suitability of assessments of attachment quality* for informing family court decision-making, concluding that such assessments should be used primarily for directing supportive interventions.
(6) We outline important questions to guide *future collaborative research* between family court professionals and researchers and attachment scholars.

Although there is consensus among us about most of these topics, there are differences of opinion on some matters. Such differences of opinion can be an asset in science and its application; a diversity of perspectives can drive the development of increasingly valid theory, research, and applications. We are careful to outline where we have different opinions, and where further research may consequently be of particular importance. Throughout, we offer our reflections in a spirit of appreciation for the challenging work of family court practice, and with the hope of contributing to further dialogue and mutual learning.

## Part I: Problems Related to the Application of Attachment Theory and Research in Child Protection and Custody Decisions

Before proposing his foundational theory of attachment, John Bowlby began his classic trilogy (1969/1982, 1973, 1980) with a heading, “observations to be explained” (p. 24). In a similar vein, Part I of this consensus statement is devoted to our observations of problems in the application of attachment theory and research in family court contexts. To help explain these observations, we address how the “best-interest-of-the-child” standard has pressed attachment theory into service. We highlight common misunderstandings that have ensued and discuss more specific factors that may have contributed to these misunderstandings.

### 1 Attachment Theory and the Best-Interest-of-the-Child Standard

The “best-interest-of-the-child” standard has become ubiquitous in family court decision-making regarding child protection and custody. However, its broad formulation has created a demand for more specific meaning to guide court practice. We discuss how the standard’s dependence on psychological theory and research has helped pull attachment theory and research into the court context.

#### 1.1 The Best-Interest-of-the-Child Standard’s Dependence on Psychological Theory and Research

The twentieth century saw the development of a child-centered approach to education and parenting. Since then, childhood is widely viewed as valuable in and of itself: children are regarded as requiring loving care to develop favourably, and there is an emphasis on parental responsibility to tend to children’s needs (Kohm, 2007). The past century and a half also witnessed the gradual emergence of the “best-interest-of-the-child” standard, initially developed in the United States but now typically associated with Article 3 of the UN Convention on the Rights of the Child (UNCRC; UN General Assembly, 1989). The UNCRC stipulates that “in all actions concerning children, whether undertaken by public or private social welfare institutions, courts of law, administrative authorities or legislative bodies, the best interests of the child shall be a primary consideration” (Article. 3, Paragraph 1). This standard is often referenced in decision-making concerning child protection and child custody. The principle has been important in countering the neglect of children’s rights and viewpoints, in itself does not favour either parent based on gender, and is aligned with a movement toward judicial discretion, giving courts freedom to weigh what may be in the best interest of each child (Schneider, 1991).

However, the principle’s broad formulation leads to a demand for more specific meaning in court practice. Specifically, the principle appears to require accounts of optimal and adequate child-rearing practices and of child development, in general, as well as in specific cases. Consequently, the principle has contributed to a need for expert assessors and witnesses with knowledge of caregiving and child development (Mnookin, 1975), and to a demand for considerations to be based on developmental theories with high scientific status.

Yet, gathering and interpreting scientific evidence has proved difficult in this context. Although best-practice guidelines have called for empirically based methods and procedures, many instruments lack sufficient validity (Emery et al., 2005). Interpretation of evidence is also inherently complicated. At times, mental health professionals make predictive claims that cannot be justified by social science research and judges may show misplaced faith in these claims (Scott & Emery, 2014). Indeed, judges can face difficulties evaluating the scientific merits of psychological methods, and courts may admit evidence with poor or unknown scientific value (Neal et al., 2019). A major challenge regarding scientific data also derives from the contrasting aspirations of science and the courts: whilst science generalises (usually from individual cases to general principles), the court particularises (sometimes from general principles back to individual cases). Thus, a common problem in court practice concerns the risk of invalid inferences for individual cases made based on trends and averages from group-level research (Faigman et al., 2016).

Determining children’s best interests is also very difficult in practice, because one must weigh the many factors that can influence children’s development, while still keeping an eye toward children’s probable future development (Salter, 2012). For instance, assessments should include factors influencing the child’s physical, cognitive, and socioemotional development, with the possibility of harm outweighing other factors. Caregivers’ ability to protect and care for their child is, of course, important for healthy child development. However, caregiving includes a variety of domains, and different domains can be differentially important for different aspects of child development. Moreover, caregiving factors can be difficult to assess objectively and may vary over time (e.g., due to temporary impact of mental health problems, drug and alcohol abuse, and environmental stressors), and their long-term implications for the child’s future development are often uncertain. As such, it has been argued that children’s best interests may be indeterminate (Mnookin, 2014) and that the principle is inconsistently applied (Emery et al., 2005; Font & Gershoff, 2020). Furthermore, family court professionals may not be able to keep track of developments in theory and research on child development, and decision-making to promote children’s best interests can therefore become influenced more by personal opinion and historical and cultural forces than by an updated understanding of the scientific evidence-base (Kelly & Lamb, 2000).

#### 1.2 The Rise of Attachment Theory in the Family Courts

The “best-interest-of the-child” standard has resulted in frequent references to attachment theory and research, and to attempts to assess attachment quality to inform decision-making concerning child protection and custody (Aitani, 2015; Crittenden & Baim, 2017; Gauthier et al., 2004). The interest in attachment theory and research may in part stem from UNCRC’s emphasis on children’s right to a family, and the importance of the family, which point to the centrality of child-parent relationships. Concern with attachment may also stem in part from attempts to operationalise the best-interest standard, which have seen the importance of child-caregiver interactions and relationships highlighted in many countries (for an overview see Skivenes & Sørsdal, 2018). For instance, the U.S. Marriage and Divorce Act includes the relationship of the child with the child’s parents as one of five factors that form the basis for judging a child’s best interests (Raub et al., 2013). However, it is typically not specified what aspects of child-caregiver interactions and relationships are most important, or how they should be assessed (Harmer & Goodman-Delhauntry, 2014). Attachment may consequently seem of special relevance in this context as reflecting the whole child-caregiver relationship, or as reflecting its most important socioemotional aspects.

The focus on child-caregiver relationships in general, and attachment theory in particular, may stem from the presumed importance of *one* “psychological parent” (e.g. a child’s principal provider of security and safety), and the relationship between the child and this caregiver, which emerged in parallel with the introduction of the best-interest discourse (for an early discussion, see Goldstein et al., 1973). From this perspective, it was extrapolated that the principal caregiver should be prioritised over other relationships, and some states in the U.S. have even mandated that the psychological parent be recognised in best-interest determinations (Jacobs, 1997).^2^ Early attachment research typically examined attachment only in relation to the parent staying at home, usually the mother, and this likely made attachment theory appear aligned with the idea of one psychological parent. The scope of subsequent attachment research may have reinforced this impression: the vast majority of studies have focused on mothers and fathers have still not been sufficiently included (Cowan & Cowan, 2019; Lux & Walper, 2019).

Another likely reason for the rise of attachment theory in family courts is that the theory – linking caregiver sensitivity to child attachment quality (Fearon & Belsky, 2016; Lucassen et al., 2011) and child attachment quality to subsequent development (Groh et al., 2017a) – has seemed to offer solid empirical ground for anchoring best-interest considerations. In sum, attachment theory has clearly provided research that can be highly pertinent for supporting children and their caregivers (Steele & Steele, 2017). Since children’s best interest is the criterion for child protection and custody decisions (Raub et al., 2013), and decisions should be empirically based and child-caregiver relationships taken into account, attachment theory and research have understandably appeared relevant for meeting the best-interest demand.

Accurate implementation of attachment theory and attachment assessments in this context has, however, been hampered by a variety of factors. First, there is a great deal of misinformation about attachment in circulation across various contexts, including the family courts. This includes misinformation about fundamental matters such as what attachment is, the nature of children’s multiple attachment relationships, and what can be inferred – at the level of an individual child – from assessments of attachment quality (Granqvist et al., 2017). In some cases, the result has been an ill-informed dismissal of the relevance of attachment by court professionals. For example, the High Court for England and Wales recently stipulated that attachment is just a statement of the obvious and based on an untenable central premise, and deemed an assessment report invoking attachment concepts inadmissible as expert evidence (GM v. Carmarthenshire County Council, 2018). In other instances, there has been overuse of attachment ideas and measures, with practice unmoored from evidence (for a discussion, see White et al., 2019).

### 2 Key Misunderstandings

Translation of research into practice depends on an accurate understanding of concepts and research findings. Regarding attachment research, however, there are a number of common misunderstandings that have hampered accurate translation into family court practice. In our view, the most important of these misunderstandings relate to: 1) the nature of attachment, 2) the interaction among multiple attachment relationships, and 3) the practical implications of classifications of attachment quality.

#### 2.1 Misunderstandings Regarding the Nature of Attachment

There are widespread misunderstandings regarding the nature of attachment, including assumptions that children are born attached; that attachment equals attachment quality; that isolated behaviours reveal attachment quality; and that attachment quality equals relationship quality, caregiver sensitivity, or “strength” of attachment.

##### 2.1.1 The Assumption that Attachment Equals Attachment Quality

*Attachment* is not the same thing as *attachment quality*, but these concepts are often conflated. Attachment refers to an affectional bond in which an individual is motivated to seek and maintain proximity to, and comfort from, particular familiar persons (Bowlby, 1969/1982). Children are born with a predisposition to develop this motivation in relation to significant others (“attachment figures”) who have been sufficiently present and responsive. For children, these persons are usually their caregivers. The motivation is held to be governed by an attachment behavioural system.This system seeks to maintain a certain degree of proximity between child and attachment figures, with the setting for desirable level changing dynamically in response to internal and external cues. The motivation to increase proximity is activated when a person is alarmed by internal cues (e.g. pain, illness) and/or external cues (e.g. fear-evoking stimuli, separation), and manifests in a tendency to seek the availability of an attachment figure. When the attachment system is strongly activated, some kind of physical contact with an attachment figure is generally sought, especially by infants, though this contact can also be achieved by non-physical means later in development. Among the most important conditions for deactivation of the attachment system is the perception that an attachment figure is accessible and responsive – able to provide a *safe haven* when the infant is alarmed. Caregivers who have regularly interacted with and protected the infant when the infant has been alarmed usually come to be represented by the infant as someone he or she can turn to when in need (i.e. as a safe haven). Importantly, even the most sensitive and responsive of caregivers necessarily “tune out” from time to time – to visit the bathroom, make tea, or even temporarily hand over caregiving to another trusted person familiar to the infant, while the caregiver attends to other matters. Thus, that a caregiver provides a safe haven does not necessitate that this person is constantly accessible for the infant physically, or even psychologically, or that the child is securely attached to that caregiver. Conversely, being physically present does not necessarily mean that a caregiver is emotionally available.

In attachment research on young children, whether an attachment relationship has developed is typically measured through observations of whether they display selectivity in directing their signals specifically toward their caregivers, particularly when alarmed.

Additional indices that an attachment relationship has been established include the child’s display of protest against involuntary separations from the caregiver, often coupled with the development of stranger wariness (Bowlby, 1969/1982).

Attachment *quality*, on the other hand, refers to variations in children’s expectations about the availability (accessibility and responsiveness) of their attachment figure in times of need (Ainsworth et al., 1978). Attachment quality presupposes that children have developed an attachment relationship in the first place, and the quality of attachment is captured by how the child’s motivation to seek and maintain the availability of their caregiver is expressed in the relationship. Almost all children form at least one attachment relationship and most form multiple attachment relationships (Posada et al., 2013); what differs is the *quality* of these attachment relationships. In attachment research, trained and certified coders capture attachment quality through standardised observations of children’s relative ability to use their caregiver as a *safe haven* to which they can turn for protection, and as a *secure base* from which they can explore the environment. Secure attachment relationships are indicated by behaviour that suggests that a child expects the attachment figure to be available in times of need, and insecure attachment by behaviour suggesting the expectation of relative unavailability.

##### 2.1.2 The Assumption that Children’s Attachment Quality Equals Caregiver Sensitivity

Children’s attachment quality is often thought to constitute a mirror image of their caregiver’s “sensitivity”; the ability to notice children’s signals, interpret them correctly, and respond to them timely and appropriately (Ainsworth et al., 1974). This perception has likely been reinforced by theory and research stressing the association between caregiver sensitivity and child attachment quality (Ainsworth et al., 1978). Indeed, the association has been replicated in many studies from numerous countries and cultures, and meta-analytic research has shown that secure child attachment is associated with more sensitive caregiving behaviour for both mothers (*r* = .24 [*d* = .49]; De Wolff & van IJzendoorn, 1997) and fathers (*r* = .12; Lucassen et al., 2011). In addition, child attachment has been found to be malleable, from insecure to secure, in interventions that result in enhanced caregiver sensitivity (Bakermans-Kranenburg et al., 2003). However, while the association between caregiver sensitivity and child attachment is significant and notable, it is small to moderate in size, and one should take caution in inferring caregiver sensitivity from children’s attachment quality. Granted, various factors can enhance measurement error in research and thereby attenuate associations (e.g. very brief observations of caregiver sensitivity). Nonetheless, other caregiving behaviours beyond sensitivity may also be important for child attachment quality. There may, for example, be a role of broader contextual factors and children’s biologically based dispositions in shaping their susceptibility to caregiving (Belsky et al., 2007). The smaller association for fathers likely reflects, in part, their comparatively lesser time spent with infants on average across samples studied to date. Moreover, while children may develop secure attachment relationships with their fathers, as with their mothers, other factors have been hypothesised to promote the development of attachment security with fathers (Grossmann et al., 2008; Zimmerman, 2017). Presumably as a result of different gender norms, measures of sensitivity and safe haven functioning may, on average, have less acuity for fathers than mothers, while measures of secure base functioning may be comparatively more important for attachment security to fathers.

##### 2.1.3 The Assumption that Attachment Quality Equals Relationship Quality

It has been argued by some (e.g., Shemmings, 2018) that the term ‘attachment’ can be mystifying and that court practitioners should substitute the word ‘relationship’ in their records and reports. The term relationship is useful in and of itself, because relationships subsume multiple domains of interaction and qualities, and family courts should want to achieve a broad view of caregiving quality. However, using relationship as a substitute for attachment has serious risks. Importantly, it risks fuelling the mistaken assumption that relationship quality and attachment quality are equivalent concepts. Attachment quality constitutes one important aspect of relationships for children, but we urge the recognition of many other important aspects of relationships, such as basic physical care, play, supervision, teaching/learning, setting standards for conduct, disciplining, and instrumental support (Zeanah et al., 2000). Attachment quality should therefore not be equated with overall relationship quality.

##### 2.1.4 The Assumption that Single Behaviours Reveal Attachment Security

Children have sometimes been precipitously assumed to be insecurely attached if they cry, or if they do not cry, in their caregiver’s presence (Bullens, 2003). Yet, attachment quality cannot be determined from isolated behaviours such as crying. First, children’s displays of attachment behaviour such as crying depend on whether they have been alarmed or not. Second, children can use different behaviours in different situations in seeking caregiver availability, depending on situational constraints. Thus, a securely attached child exposed to a threatening noise may cry in order to increase proximity to its caregiver when seated in a high chair, but approach the caregiver to receive comfort (with or without crying) when freely able to move. In addition, isolated behaviour such as crying can depend on other factors besides attachment. For instance, whether or not children become distressed is related to individual differences in temperament (i.e. biologically grounded individual differences in reactivity and regulation; Belsky & Rovine, 1987; Groh et al., 2017b). In assessments of attachment quality, a careful examination is therefore made of how various behaviours relate to one another in the service of using the caregiver as a safe haven and secure base, with due concern for the context of these behaviours (Ainsworth et al., 1978).

##### 2.1.5 The Assumption that Children are Born Attached

Children are born with the capacity for care-seeking behaviour and a predisposition to form attachment relationships. However, attachment relationships are built over time, through recurrent interactions with caregivers, and depend on the opportunity to develop expectations regarding the attachment figure’s accessibility and responsiveness. In fact, attachment relationships are typically observed only from the last quarter of children’s first year of life. Before that, it is unquestionably possible to assess aspects of caregiving, for instance caregiving sensitivity (Pederson & Moran, 1995). However, it is currently not advisable to assess children’s attachment quality until the age of about one.

##### 2.1.6 The Assumption that Attachment Quality Equals Strength of Attachment

Insecure attachment is sometimes inaccurately characterised as “weak” attachment (for a discussion, see Schofield & Walsh, 2010). Human children are very vulnerable and depend on their caregiver’s support for a long time, and the capacity to develop attachment relationships appears to be universal in humans (Bowlby, 1969/1982; Mesman et al., 2016). In fact, children develop attachment relationships even if their caregivers are rejecting, inconsistently sensitive, or abusive (Simpson & Belsky, 2016). Furthermore, though some types of attachment relationships are termed “insecure”, they are nonetheless regarded as adaptive strategies for children that may maximise the potential availability of a caregiver (Main, 1990). Moreover, an insecure attachment relationship does not mean that the caregiver is never a safe haven for the child.

For these reasons, references to strong or weak attachment as equivalent to secure and insecure attachment are misguided. In fact, some children from insecure attachment relationships display strong attempts to seek a familiar caregiver, mixed with anger toward the caregiver. Furthermore, some children from secure attachment relationships make little attempt to seek their caregiver, even when moderately alarmed, since they are confident in the caregiver’s availability. The terms “strong” and “weak” are therefore generally not used by attachment researchers, and never when referring to children who have had sufficient time and interaction with a caregiver to develop attachment. Absence of attachment to caregivers is extremely rare and typically observed only among children who have had insufficient time to develop attachment relationships (e.g., due to recent placement in a new caregiving arrangement, like foster care) or among the very few children who have not had sufficient opportunities to identify any caregiver as familiar (e.g., due to institutional rearing; Zeanah et al., 2005).

#### 2.2 Misunderstandings Regarding the Interaction Among Multiple Attachment Relationships

In this section, we discuss misunderstandings regarding the importance of developing attachment to one particular caregiver (“the psychological parent”), rather than to more than one. We address how misunderstandings on this matter have been expressed following parental divorce, potentially influencing both (1) custody decisions and (2) overnight arrangements. We argue that for the courts to reach legitimate decisions on these matters, they must attend to a given child’s developmental context.

##### 2.2.1 Multiple Attachment Relationships and Custody Decisions

It is often assumed that an attachment relationship with one person is at the expense of other attachment relationships, and that best-interest decisions should maximise the likelihood of secure attachment with one “primary caregiver”. For instance, custody decisions have been characterised as “balancing the benefit of a secure attachment to one parent against the benefit of forming attachments to both parents” (Tornello et al., 2013, p. 871). However, children can develop and maintain secure attachment relationships to multiple caregivers simultaneously, if they have sufficient time together and the caregivers respond in ways that provide a safe haven for the child in times of need (Kelly & Lamb, 2000). While we currently do not know how much time is needed for development and maintenance of attachment relationships, decisions to categorically prioritise one parent may hamper children’s opportunities to form and retain attachments to other caregivers.

Notably, whereas little contact between young children and their non-custodial caregivers (typically fathers) is predictive of little or no contact also in the future (Cheadle et al., 2010), joint physical custody is associated with more enduring relationships with the non-resident parent (Steinbach, 2019). However, as noted by Steinbach (2019), most of the research that has found positive effects of joint physical custody has been in the contexts of low inter-parental conflict and with older children drawn from families with high socio-economic status. Research in additional contexts is consequently needed. Yet, with the increase in the time and range of father involvement in childrearing world-wide, studies have pointed to the beneficial and distinct effects of the father on the child's neurobiological maturation (Feldman et al., 2019), and on the development of social competencies, particularly the child’s capacity to manage aggression (Bacro & Macario de Medeiros, 2020; Feldman et al., 2013). Thus, depriving children opportunities for relationships with their fathers is generally not in their best interest. In fact, even in traditional families with low to moderate father involvement, long-term studies have shown positive effects of paternal sensitivity on child development (Grossmann et al., 2008).

Early claims by Bowlby indicated that one primary relationship is of special importance. However, Bowlby later changed his mind in this regard (Bowlby, 1984), and indeed, it has not been the position among the vast majority of attachment researchers for decades (Duschinsky, 2020). In fact, attachment researchers generally hold that humans evolved with the expectation of a limited network of attachment relationships with particular, familiar people who can be turned to in times of need (Abraham & Feldman, 2018; van IJzendoorn, 2005). This multiple caregiver phenomenon is indeed the norm in many cultural settings (Hrdy, 2011). Multiple caregivers and a network of attachment relationships have also been found to constitute a protective factor in child development, with secure attachment to one person buffering the implications of insecurity in other relationships (Bacro et al., 2020; Boldt et al., 2014; Egeland et al., 1988; Saunders et al., 2011; van IJzendoorn et al., 1992). Relatedly, in cultures where extended-family dwelling is the norm, children not only benefit from multiple extraparental attachments with kin, but such attachments may mitigate some of the difficulties observed in the mother-child relationship, such as when the mother is depressed (Feldman & Masalha, 2007). Thus, convergent evidence suggests that each attachment relationship is important, with children gaining benefits from having more than one safe haven (Dagan & Sagi-Schwartz, 2018).

It would be a mistake to infer from this discussion that a child can form countless attachments of equivalent significance; there are certainly limits, even if not well specified (van IJzendoorn et al., 2020). Furthermore, the child (especially younger children) may still prefer some caregiver(s) over others when it comes to meeting attachment needs (Bacro et al., 2020). Nonetheless, the psychological and developmental meaning of such a preference is not self-evident. For instance, this preference is typically only seen when more than one caregiver is currently accessible, and it does not seem to depend on attachment quality to the respective parents (Umemura et al., 2013; Zimmerman, 2017). In addition, children’s preferences in the moment may depend on contextual factors (Lamb, 2018). Yet, it should be noted that there is currently not enough scientific knowledge about children’s preferences in contexts of inter-parental conflict and custody disputes.

Based on the concerted body of research, most attachment researchers agree that children’s attachment relationships with all their regular caregivers are important and should be supported. What differs among attachment researchers – the current authors included – is whether the relationship with a “most familiar” caregiver may have particular importance as a safe haven in the earliest years of child life, and whether this caregiver – in the context of custody decisions – should consequently be allocated more time with the child until the child’s cognitive development makes separations from the most familiar caregiver more tolerable (e.g. Main et al., 2011; Sroufe & McIntosh, 2011; this position has been criticised by Lamb, 2012, 2018). Yet, current research is too scarce for a definitive and straightforward empirical answer to this question. This is partly because the answer likely differs depending on context, like culture (e.g. predominantly individualistic, interdependent, or collectivistic orientations), familial factors (e.g. equal or unequal division of caregiving responsibilities between spouses, inter-parental conflict post-divorce), and children’s development (e.g. infants/toddlers vs somewhat older children). We urge court practitioners to consider such pertinent contextual circumstances in settling custody arrangements for children, but to strive for continuous contact with both caregivers wherever possible.

When a “second” caregiver – for whatever reason – has been markedly uninvolved in caregiving and other forms of interaction with the child pre-divorce, it is important for the child to have the opportunity to become gradually adjusted to being cared for by that caregiver post-divorce, before he or she is allocated fairly equal time for caregiving responsibilities (Kelly & Lamb, 2000, 2003; Warshak, 2014). This is especially true for infants and young toddlers who are just about to form or have just formed selective attachments to the caregiver(s) with whom they have had a continuous interaction history. Notably, this discussion pertains specifically to safe haven provision, and not to other aspects of the relationship. As noted by Main and colleagues (2011), a non-resident caregiver can fulfil other important relationship functions for the child (e.g. playful interaction), so having regular contact with this caregiver usually serves the child’s development well beyond the gradual adjustment to being cared for by him or her.

It is unfortunate that attachment theory and research is sometimes regarded as yielding blanket support for one form of custody arrangement over all other ones. Sometimes, the theory has been characterised as supporting an emphasis on one psychological parent, typically the mother. In other cases, the theory has been held to categorically prescribe joint physical custody, with equal time allocation regardless of child age, including transitions between family homes every day or every other day. One particular instance of the former can be found in the Tender Years Doctrine, in which custody automatically goes to the mother for children under a certain “tender” age. Whilst having been formally replaced by the best interests of the child standard in most countries, it has been argued that the Tender Years Doctrine continues to influence child custody decision-making (for discussion, see Artis, 2004). Also, it is still in active use in some countries, whether or not referencing attachment theory (Aitani, 2015; The National People’s Congress of the People’s Republic of China, 2020).

One such country is Israel, where custody automatically goes to the mother for children under the age of six, except under very special circumstances when the mother is deemed unfit. In Israel, the Doctrine has been defended by influential voices in the field of law, supported by misinformed references to attachment theory (Hacker & Halperin Kaddari, 2013). In response to those who have argued that equal parental responsibility is implied by attachment theory and research (Joels & Sagi-Schwartz, 2012), Hacker and Halperin Kaddari (2013) referenced a special issue of *Family Court Review* with contributions from a selected group of attachment scholars (McIntosh, 2011). They argued that there is general consensus that infants form a primary attachment with one caregiver and that parenting arrangements in divorce situations should reflect this consensus (for discussion, see Warshak, 2014, vs McIntosh et al., 2015). As discussed above, that does not represent a consensus view. More importantly, however, we are in full consensus that the ultimate establishment of a network of attachment relationships is generally a protective factor in the long term and thus a desirable outcome in child development. We are also in full agreement that losses of and permanent separations from attachment figures are in themselves risk factors that should be prevented wherever possible in child development.

##### 2.2.2 Multiple Attachment Relationships and Overnight Arrangements

A related issue is the argument that overnight care with non-resident caregivers is inherently harmful for younger children and should be discouraged within custody arrangements. Such claims stem in part from overreliance on an early and misinterpreted study by Solomon and George (1999). The authors concluded that co-parenting arrangements that included overnight visits to the co-parent were associated with attachment insecurity with the resident parent. However, their own data actually indicated a non-significant difference and that parental conflict was a better predictor of insecurity (van IJzendoorn et al., 2019; see also Lamb, 2018). Solomon (2013) has subsequently criticised the use of their study to argue against overnights with non-resident parents. In addition, most current evidence suggests no negative effects on attachment security (Lamb, 2018; see also Fabricius & Suh, 2017). Nonetheless, the Solomon and George (1999) study is often referenced to demonstrate the potential dangers of overnight visits to the non-resident parent (e.g. Tornello et al., 2013; McIntosh et al. 2013; for a discussion see Pruett et al., 2016).

Related to the issue of how infants spend the night, a study conducted in Israeli kibbutzim (Sagi et al., 1994) found that overnights with unfamiliar watch-women in collective sleep arrangements were associated with high rates of attachment insecurity with mothers. While these findings are important, they do not speak to the issue of whether overnights with non-resident parents have adverse effects on attachment relationships with resident parents. More specifically, the kibbutz study implied that overnight arrangements in which children do not have access to any familiar safe haven at all might have negative impact on attachment security, due to negative effects on children’s expectations regarding their attachment figures’ availability.

Purely from an attachment perspective, some among us hypothesise that home is where the child’s familiar caregivers are, and further hypothesise that attachment security in children who have been regularly cared for by both caregivers pre-divorce are unlikely to be hampered from overnights with any of these caregivers post-divorce, regardless of child age. Others among us hypothesise that whether overnights with a non-resident parent have adverse developmental effects is likely to depend on the same set of contextual factors we discussed above (i.e. developmental, familial, cultural). Though there is no scientific literature on which to base exact age-related recommendations, we consensually hypothesise that overnights should be unproblematic from preschool age onwards, if both caregivers have cared for the child regularly pre-divorce. In contrast, an infant or young toddler who has had very little opportunity to develop safe-haven expectations in relation to one of the caregivers may find it more difficult, at least initially, to spend overnights with that caregiver post-divorce. Of course, children may also experience initial difficulties related to other factors than insufficiently developed safe-haven expectations, such as unfamiliarity with a caregiver’s new physical setting. However, children who have developed clear safe-haven expectations to both caregivers are likely to adjust relatively quickly and cope well with overnights in both homes. Yet, further research is needed to establish what degree of familiarity is required for children to sense they have a safe haven available when spending the night with a non-resident caregiver – or for that matter any other caregiver in an attachment network.

Many attachment researchers believe that physical custody by and overnights with a given caregiver may facilitate the child’s development of an attachment relationship with that caregiver (Lamb et al., 1997). One reason is that children’s attachment system is thought to be complemented by a caregiving system in caregivers, which – similar to the child’s attachment system – is malleable and open to input from the environment (George & Solomon, 2008). Seriously depriving a caregiver of time with his/her child and caregiving responsibilities may consequently not only influence the child’s ability to develop and maintain an attachment relationship to the caregiver. It may also have untoward effects on the caregiver’s caregiving system, which may become thwarted. However there is no empirical research suggesting that overnights are essential (i.e. a necessary condition) for the development of an attachment relationship.

Finally, decision-making regarding child custody and time allocation, including sleeping arrangements, should also take into account caregivers’ ability to cooperate post-divorce. In some countries, attachment theory is invoked to categorically motivate joint physical custody, with little regard to contextual factors such as inter-parental conflict and ability to cooperate. Post-divorce interparental conflict has been linked to a variety of negative effects on child adjustment (Tan et al., 2018; van IJzendoorn, 2019), including on child attachment (Brown et al., 2010; Solomon & George, 1999). Interparental conflict and hostility may not only undermine one’s own parenting competencies but also one’s ability to let the other provide care (Grossmann, 2013), with negative ramifications for the child who is caught in the middle. Interventions to support caregivers’ involvement and decrease interparental conflict post-divorce have been developed to address such difficulties and shown promising results (Pruett et al., 2016).

#### 2.3 The Implications of Classifications of Attachment Quality

Attachment classifications are often misunderstood and misused in applied contexts, and we recognise that the community of attachment researchers, including many of us, have at times inadvertently contributed to this situation (Duschinsky, 2020). We have occasionally advocated our methods to assess attachment quality and exaggerated the implications of pertinent findings without explicitly acknowledging their constraints and limitations. In hindsight, it is plain to see that we should have been more careful. Measures to assess attachment quality, when used by trained and certified coders in validated settings, are impressive tools for research on the *group-level*. Yet, questions arise regarding the transferability of the measures’ validity to family court settings and *individual* children (and caregivers). The key question here is of course whether assessments of attachment quality yield useful information to inform decision-making regarding child custody and child protection. Valid information about an individual child’s attachment quality can yield valuable insight into that child’s relationship with a given caregiver and could conceivably increase professionals’ ability to predict that child’s probable development. However, and as discussed below, the effect sizes for the associations among children’s attachment quality, pertinent caregiving behaviour, and subsequent child development are small to moderate. Consequently, attachment measures do not have sufficient predictive power to serve as stand-alone “proxies” for individual children’s caregiving history or how they will develop.

##### 2.3.1 The Assumption that Attachment Classifications Provide Reliable and Valid Information about Individual Children’s Caregiving History and Developmental Prospects

Attempts to assess attachment quality are occasionally made to inform caregiving assessments and family court decision-making regarding child protection and child custody (Aitani, 2015; Crittenden & Baim, 2017; Gauthier et al., 2004). Attachment measures have, however, been developed and validated for group-level research, and validity for group-level research does not automatically transfer to sufficient validity for individual-level diagnostics or prediction (Neal et al., 2019; van IJzendoorn et al., 2018a). In medical science and clinical settings, diagnostic instruments must have high “sensitivity” and “specificity” to be considered useful. Whereas *sensitivity* refers to the proportion of “true positives” that are correctly identified (e.g. securely attached children who are correctly classified as securely attached), *specificity* refers to the proportion of “true negatives” (e.g. insecurely attached children who are correctly identified as not securely attached). Tests can have both high sensitivity and specificity, although a test with high sensitivity may have lowered specificity if it yields many “false positives” (e.g. identifying most securely attached children as securely attached, but also identifying many insecurely attached children as securely attached). Regarding attachment assessments in the current context, sensitivity and specificity considerations may be extended to the instruments’ retrodictive and predictive ability; for example, to identify securely attached children who have experienced sensitive caregiving and who develop favourably, as well as insecurely attached children who have not experienced sensitive caregiving and who do not develop favourably.

Few psychological – or for that matter biomedical - instruments developed for group-level research have sufficient sensitivity and specificity for valid diagnostic use and prediction of development at the individual level (Neal et al., 2019). Problems with psychometric precision and predictive power are perhaps particularly notable for assessments in infancy. Attachment assessments are in fact among the most powerful measures in infancy in predicting subsequent child development in *group-level* research (Groh et al., 2017a). That most psychological instruments have insufficient predictive power on their own should not be surprising; human development is truly complex so no instrument should be expected to explain most of the variance in developmental outcomes. It is nonetheless important to note that current attachment measures have limited sensitivity and specificity for predicting individual children’s development or retrodicting individual children’s caregiving (van IJzendoorn et al., 2018a). In particular, a high proportion of children classified as insecurely attached develop favourably and have experienced at least sufficient (if not consistently sensitive) care.

The predictive ability of attachment measures on the *group-level*, together with their limited sensitivity and specificity for *individual-level* prediction, has contributed to different opinions among us attachment researchers regarding their usefulness for informing family court decision-making, particularly regarding child protection. Some among us would want to see higher validity (especially sensitivity and specificity) for individual level prediction before supporting the use of these measures for informing decision-making regarding out-of-home placement. Others among us believe that attachment measures can be helpful as contributors to “the larger picture” if used in conjunction with other measures. These differences in opinion, which we further discuss below, partly depend on different views regarding how high the standards should be for a scientific instrument to be deemed useful in informing family-court decision-making.

##### 2.3.2 The Assumption that Secure Attachment Equals Psychosocial Health, Forecasts Individual-Level Psychosocial Health, and Provides an Index of a Child’s Best Interests

Meta-analytic research has shown that secure attachment in childhood is subsequently associated with greater social competence (*d* = .39) and lower externalising (*d* = .31) and internalising problems (*d* = .15; Groh et al., 2017a). These effect sizes are notable, and have relevance in terms of effects averaged over many children and studies (Funder & Ozer, 2019). Secure attachment is generally a protective factor in human development (Scott et al., 2011), and it follows that policies and interventions that facilitate sensitive caregiving and secure attachment have practical utility (Bachmann et al., 2019). These effect sizes might also be seen as pragmatically adequate for motivating the use of measures of attachment security in family courts, especially given that many other available instruments have lower or unknown predictive validity. Validated attachment assessments, if conducted by trained and certified coders, may yield information that increases professionals’ ability to predict children’s probable future development at least above chance levels. However, the effects are not of a size to imply that a child’s future development can be predicted with confidence solely from assessments of attachment security. If courts use attachment assessments to inform decisions, the weight assigned to the attachment classifications must reflect this predicament.

Communicating the complexities involved in translating group-level research into assessment of individual children and caregivers has proven difficult, with the importance of secure attachment often exaggerated or communicated in other misleading ways in social work guidelines. Such characterisations can prompt or sustain misguided perceptions that secure attachment is necessary for favourable child development. In turn, this can contribute to an overemphasis on secure attachment in family court decision-making. An example of unclear communication can be found in UK Department of Health practice guidance that, whilst no longer statutory, has been and remains very influential: “What happens to children in the first years of life is the foundation of later development and will affect their outcomes. The significance of this must be taken into account in the assessment process. This is why secure attachments are so important in the early years. Where these attachments are absent or broken, decisions to provide children with new attachment figures must be taken as quickly as possible to avoid developmental damage” (Department of Health, 2000, p. 3).

This guidance is unclear and misleading because it begins by advancing the importance of secure attachment; then creates an extreme contrast between secure attachment and “absence” of attachment and “broken” attachments; and then argues for quickly providing children with new attachment figures. The deterministic tone neglects the fact that later experiences also influence attachment relationships (e.g. Waters et al., 2000). Moreover, in the Department of Health practice guidance there are no grey areas; no mention of insecure attachment, nor any reference to how rare lack of attachment is. Presumably far from the authors’ intentions, this guidance may have prompted or reinforced perceptions that anything but secure attachment is associated with a high risk for unfavourable development, and that children should be considered for removal from their caregivers in the absence of secure attachment (White et al., 2019). Such perceptions would constitute a grave misunderstanding of attachment theory and research, while implying that almost half of all children should be taken from their parents – as this is the average rate of insecure attachment in the general population (van IJzendoorn et al., 1999).

##### 2.3.3 The Assumption that Organised Insecure Attachment Implies Harm and Pathology

Two kinds of “organised” insecure attachment have been distinguished, as assessed by trained coders in a separation-reunion assessment known as the Strange Situation Procedure (Ainsworth et al., 1978). In insecure-avoidant attachment relationships, children do not seek their familiar person when mildly alarmed, although the child remains near. In insecure-resistant attachment, children do seek proximity, but are not readily comforted, and mix proximity-seeking with displays of anger toward the caregiver. These patterns of minimising and maximising signals of attachment needs are considered “organised” because behaviour is coherent and may function to increase the availability of less sensitive caregivers. Meta-analyses have revealed significant and robust but modest associations between avoidant attachment and lower social competence (*d* = .17), higher levels of internalising problems (*d* = .17), and higher levels of externalising problems (*d* = .12); and between resistant attachment and lower social competence (*d* = .29; Groh et al., 2017a).

Such effect sizes do not imply that organised insecure attachment in and of itself can be used as a proxy for inadequate care or to forecast unfavourable child development. While the effect sizes suggest that insecure attachment, if validly assessed, can contribute to weakly forecasting probable future child development, one can legitimately question how much weight to place on this forecast and its practical significance. The effects of insecure attachment are also dwarfed by the adverse effects associated with absence of opportunities to form attachments with familiar caregivers, as seen in institutionalised children (van IJzendoorn et al., 2020). Indeed, the capacity for diverse (including insecure) patterns of attachment likely evolved by contributing to child survival and adaptation to varying caregiving and contextual conditions (Belsky, 1997). Unless the world is successfully engineered to become a responsive and safe place with plenty of resources for all of its inhabitants, it may not be justified to consider only secure attachment relationships to be adaptive for all individuals. Finally, attachment quality always interacts with other factors conveying risk and protection. For instance, being in an insecure attachment relationship may have differential importance depending on factors such as quality of day care, family social support, and child temperament (van IJzendoorn & Bakermans-Kranenburg, 2012), all of which may increase or attenuate risk.

##### 2.3.4 The Assumption that Insecure-Disorganised Attachment Invariably Implies Harm and Psychopathology

Disorganised attachment is a third insecure category, identified by trained coders on the basis of conflicted, confused, or apprehensive behaviours towards a familiar caregiver under standardised conditions of mild/moderate alarm (Main & Solomon, 1986). Disorganised attachment is predicted by frightening, frightened, and dissociative caregiver behaviour (Main & Hesse, 1990; Schuengel et al., 1999), by atypical caregiving behaviour such as hostility and withdrawal (Lyons-Ruth & Jacobvitz, 2016), and by maltreatment (Carlson et al., 1989; Cyr et al., 2010). Further, meta-analytic research of the distribution of attachment classifications among institutionalised children have found secure attachment in less than a fifth, and disorganised attachment in more than half (Lionetti et al., 2015; van IJzendoorn et al., 2020).

The link between disorganised attachment and maltreatment has led some social work academics to recommend the use of disorganised attachment as an indicator of maltreatment in child protection practice (Shemmings & Shemmings, 2011; Wilkins, 2012 [but see Wilkins 2020]). The problem is that even if children who are maltreated are markedly more likely than other children to develop disorganised attachment relationships (around 50% of maltreated children do; van IJzendoorn et al., 1999), a notable proportion of maltreated children do not. Likewise, a significant proportion of children in community samples (10-15%), many of whom have not been subjected to maltreatment, display disorganised attachment during the strange situation (van IJzendoorn et al, 1999). Importantly, there are many pathways to disorganised attachment, several of which do not include maltreatment (Bernier & Meins, 2008; Solomon et al., 2017). As proposed by Main and Hesse (1990), caregivers may for instance show subtle disorganising frightening/frightened behaviours in the absence of maltreatment, due to unresolved trauma stemming from the caregiver’s own experiences of loss or abuse (Jacobvitz et al., 2006; Madigan et al., 2006). Meta-analytic research has found that infants whose families experience five or more socioeconomic risk factors have statistically indistinguishable rates of disorganised attachment as infants from maltreatment samples (Cyr et al., 2010). Disorganised attachment may also be more prevalent following major separations, as can happen during divorce and custody processes, especially in the context of acrimonious handovers and visitations (Solomon & George, 2011).

Children can also display disorganised *behaviour* without this signifying disorganised attachment and its associated relational history. Many normally “organised” children will express disorganised behaviour if stressed enough. Consequently, overstress during attachment assessments may result in displays of disorganised behaviour that do not reflect disorganised attachment or a disorganising relational history (Granqvist et al., 2016). Self-regulatory difficulties in newborns (Padrón et al., 2014; Spangler et al., 1996) and ADHD-symptoms in children have also been associated with disorganised behaviour/classifications, and may represent “false positives” for a variety of reasons (Forslund et al., 2019).

Notably, group-level research has established disorganised attachment in infancy as one of the strongest predictors of subsequent child development. For example, the meta-analytic association between disorganised attachment and externalising behaviour problems (*d* = .34) is clearly not trivial. Disorganised attachment is consequently a relevant phenomenon that should not be neglected. However, the associations between disorganised attachment and risk for adverse outcomes (externalising behaviour problems included) are still not sufficiently strong for disorganised attachment per se to be taken as an indication of pathology in family court decision-making regarding individual cases (Fearon et al., 2010). In recognition of the complexity surrounding the phenomenon of disorganised attachment and its etiology, some scholars have revised their previous positions and now emphasise that disorganised attachment is not in and of itself an indicator of maltreatment (Wilkins, 2020).

##### 2.3.5 The Assumption that Insecure or Disorganised Attachment Signifies Attachment Disorder

The term ‘attachment disorder’ has at times been used in court practice to mean “problematic attachment”, which in turn vaguely connotes attachment relationships that are not in children’s best interests (White et al., 2019). However, ‘attachment disorder’ is a technical term, and wholly distinct in meaning from insecure or disorganised attachment. It originates in the American Psychiatric Association’s Diagnostic and Statistical Manual (DSM, currently in its fifth edition), and refers to two specific and very rare conditions that are most typically seen in children who have been institutionalised (Zeanah et al., 2005). The first condition, “Reactive Attachment Disorder (RAD)”, is characterised by the child showing a lack of care-seeking towards any caregiver when alarmed, and can only be diagnosed if there has been extremely inadequate caregiving and if symptoms have started after the age of 9 months and before 5 years of age (Zeanah et al., 2016). The second condition, “Disinhibited Social Engagement Disorder”, is characterised by the child being socially non-selective and overly friendly toward unfamiliar people (Zeanah et al., 2016).

Widespread overuse of attachment disorder diagnoses, as well as overuse of the term ‘attachment disorder’ in the absence of diagnosis, have been documented (Allen & Schuengel, 2020; Woolgar & Baldock, 2015). For the vast majority of children with Reactive Attachment Disorder, symptoms disappear when placed in a stable caregiving environment that enables development of selective attachment relationships (Smyke et al., 2012), and which support caregivers’ emotional availability (Barone et al., 2019). It should be noted that “attachment therapies” for attachment disorders are circulating, which are claimed to be effective but actually have no scientific evidence-base (Allen, 2018; Chaffin et al., 2006; Mercer, 2019). This is in stark contrast to attachment-based intervention and prevention programs for disorganised attachment, which have shown robust positive effects (Bernard et al., 2012; Facompré et al., 2018).

### 3 Factors Contributing to Misunderstandings in the Translation of Attachment Research into Court Practice

Court practitioners have recognised a discrepancy between the promises that have sometimes been made about the relevance of attachment theory, and the reality of its relevance for their work (Robertson & Broadhurst, 2019). Although social workers generally regard attachment research as potentially valuable, they often lack confidence in linking attachment principles to particular cases, and worry that judges and lawyers may react sceptically to claims of expertise regarding attachment (Duschinsky, 2020; North, 2019). There is, critically, little formalised infrastructure to help practitioners match attachment considerations to court needs and to support research-practice links. This is in contrast to the field of medicine, where infrastructures include, for example, clinical diagnoses that are mutually accepted, protocols for assessment practice, funding for research on the topic, and fellowships to help clinicians become researcher-clinicians. Beyond this general point, we have identified seven additional specific factors that we suspect have contributed to problems of translation into court practice and which have hitherto generated insufficient attention and interest (an exception is Garber, 2009).

#### 3.1 The Use of Scientific “Evidence”

Scientific evidence is sometimes referenced in best-interest assessments with individual studies being assigned inappropriate weight (Nielsen, 2014). Although superior to anecdotal or non-scientific evidence (e.g., an attachment evaluator “just knows” that a particular parent is abusive), individual studies have high risks of false positives and false negatives, particularly when sample sizes are small, as is often the case for research on applied topics such as attachment. Research evidence should be treated as credible when multiple high quality studies point to the same conclusion, particularly when conducted by different research groups. Caution is still warranted though, since unless the literature is analysed rigorously, perceived convergence may stem from contrary or complex results being ignored or downplayed. Unfortunately, guidance for practitioners have sometimes cherry-picked and focused on the studies of disorganised attachment with the strongest results (e.g. Brown & Ward, 2013), rather than taking a representative account of existing research.

Meta-analyses are therefore important, because they comprise a systematic search for all relevant studies, followed by statistical analyses to calculate average effects. They can answer questions of whether there are in fact replicable associations between variables, how strong these associations are, and if they are influenced by other variables. Very large meta-analytic effect sizes can be viewed as indicating a clearly increased probability that the effects may be applied to individual cases, but meta-analyses also have limitations: the results are still on the group level, and very large effect sizes are rarely found, typically rendering effect sizes difficult to extrapolate to individual cases (Funder & Ozer, 2019). Moreover, there is sometimes reason to suspect a publication bias against null findings; even meta-analytic results may be inflated (Kvarven et al., 2019). Evidence is therefore strengthened when meta-analytic results are supported by large sample evidence as well as experimental research, such as randomised control trials of interventions, which allow for temporal-ordered evidence and causal inferences (van IJzendoorn et al., 2020).

Admittedly, the high scientific “ideals” outlined above may often be difficult to implement consistently in court practice. There may be, for instance, a lack of meta-analyses and/or randomised controlled trials on the relevant topics. Indeed, the attachment research community has not done enough research on topics and with samples relevant to court practice (e.g., time-allocation, overnights, and inter-parental conflict in relation to child attachment). Rather than precluding any application of attachment research, professionals must then draw responsible conclusions from the available research, by identifying high-quality studies and patterns on which such studies converge. There is also likely to be no group of studies that can ever satisfy all of the particulars of an individual custody or child protection dispute. That is, at some point, experts and judges may have to move from the general to the particular, and sometimes even beyond extant data. The above discussion is not meant to preclude any application of attachment theory unless there are meta-analyses with large effect sizes supported by randomised controlled trials. We do urge, however, that courts and the experts that they consult remain attentive to the true state of scientific evidence, which should inform how heavily such evidence is weighted.

#### 3.2 The Pressure for Decisions to Appear Evidence-Based and the Need for Psychological Expertise

While courts must be pragmatic with their available funding and time, there is growing pressure for decisions to appear evidence-based, justifiable, and auditable (Huntington, 2018). Because best-interest decisions are about children’s probable futures, prognosis by psychological experts is attractive. As noted, this has paved the way for attachment theory to enter the courtroom (Mnookin, 2014). Indeed, attachment theory has been found to be by far the most popular theory among professionals working with children and families in need of support (Department for Education, UK, 2018). Moreover, whilst the task of judges, who have discretion and are in dominant positions, is to determine the facts, they depend on experts who link credible sources of knowledge to children’s situations and probable futures (Schneider, 1991). It may be suspected that the pressure to find evidence relevant to best interest assessments has contributed to a short-circuit between concerns of attachment quality and best-interest assessments, and to overconfidence in the prognostic value of attachment classifications in individual cases.

#### 3.3 Popularised Accounts of Attachment Theory

Simplified accounts of attachment theory that rely on the everyday connotations of terms like ‘security’, ‘disorganisation’ and ‘attachment’ have at times been circulated in guidelines for social workers and court professionals. For instance, disorganised attachment is often mischaracterised as feelings of danger and psychological abandonment in relation to a caregiver, and as strongly prognostic for later mental illness (e.g. Brown & Ward, 2013). Similarly, a Swedish child rights agency writes alarmingly about child disorganisation as a “serious” risk factor for externalising behaviour problems, noting “… fear exists in the relationship between caretaker and child. The child is frightened by the caregiver, or the carer is afraid of the child” (Barnombudsmannen, 2007, p. 84). Such descriptions would systematically misidentify disorganised attachment (Granqvist et al., 2017). Similarly, guidance for Chilean professionals working within child protection (Departamento de Protección de Derechos Servicio Nacional de Menores, 2019) suggest the usage of an instrument for caregiving assessment that makes references to “healthy attachment” and which conflates disorganised attachment with attachment disorder (Barudy & Dantagnan, 2010). Such descriptions appear shaped more by cultural presentations of mental health and illness, the latter as chaos and unpredictability, than by an actual understanding of attachment, including disorganised attachment (Reijman et al., 2018). Scientific practice is of course always shaped by social values to an extent, but popularised accounts of attachment theory have been especially vulnerable to presenting social values as scientific facts, as exemplified by value-judgements about the roles of mothers and fathers (for a discussion, see Duschinsky, 2020).

#### 3.4 Unmooring of Academic Constructs from their Caveats

Academic constructs are sometimes unmoored from their caveats when passing into court practice (Nielsen, 2014), and this can lead to overconfidence about the implications of individual differences in attachment quality. As an example, the “Attachment Styles Interview” (ASI; Bifulco et al., 2008) is sometimes used for determining suitability to provide foster care. As discussed by Granqvist (2016), this measure has not been sufficiently validated for assessing caregiving capacity, and the authors of the measure state as much. Yet, this caveat has slipped away from court practice, as seen in a recent Swedish case in which a pair of twins were removed from their intended permanent foster home, after a year in their care, on the sole basis of the foster parents’ “insecure” ASIs (Bunnvik, 2016). In fact, the foster parents fared well on all other assessments and the children seemed to be developing well. To repeat an example from child protection practice, scholars have exaggerated the overlap between maltreatment and disorganised attachment, sanctioning for social workers to identify disorganised behaviours, in naturalistic settings and without reliability training, as an indicator of maltreatment (Shemmings & Shemmings, 2011).

#### 3.5 The Credibility of Attachment Classifications

Attachment classifications originate in developmental science and are rightly reputable within this context. However, there have at times been insufficient recognition of the need for training to properly assign them, understand their meaning, and ensure their appropriate usage. This unfortunate combination has likely contributed to the popularity of attachment classifications in child welfare professions. For instance, the Swedish National Board of Health and Welfare (2018a, b) instructs professionals, most of whom lack formal training in attachment theory and assessment, to attend to potential signs of insecurity and attachment problems, exemplified by children being anxious and clingy and wanting to know where their parents are and what they do. With such imprecise, broad, and seriously problematic descriptions, a wide range of behaviours may be misidentified as insecure.

Although the basics of attachment theory are typically a mandatory part of professionals’ training, specialist training in *assessing* attachment quality is not. Some court professionals therefore lack confidence in the relevance of attachment theory and disregard the theory’s potential; others appeal overconfidently to attachment classifications in their assessments; others seek specialist training (North, 2019). While attachment instruments were not developed for diagnostic research and have yet to be validated for such purposes, seeking specialist training may still represent the best of these three positions. Specialist training generally also transfers knowledge about the instruments’ limitations (e.g. their limited capacity to provide diagnostics and prediction at the individual level). Without infrastructure to effectively link research and practice, such as diagnostically validated assessments, practitioners who feel that making classifications of attachment would help inform their work presently face an impasse.

#### 3.6 Mistaking Advocacy for Balanced Evaluation

Advocacy can sometimes cause problems when it is mistaken for, or framed as, balanced evaluation or scientific consensus (Emery et al., 2016). Such examples can be found in the heated debate regarding child attachment, custody decisions, and overnights, as previously discussed. A special issue of *Family Court Review* (McIntosh, 2011) spurred that debate. In that issue, a small group of well-known attachment scholars were asked to comment on how attachment theory could be used, resulting in some incautious claims. For instance, some argued that science suggests that one primary caregiver needs to be the constant source of bedtime routines (Schore & McIntosh, 2011). As discussed above, convergent scientific evidence does not support this (Lamb, 2012, 2018). Appeals to attachment in the family courts would likely be less partial, more balanced, and more aligned with convergent evidence if the court called in the experts rather than the representing parties (e.g. lawyers), for whom winning the case may sometimes obscure a focus on the best interest of the child.

#### 3.7 The Evidentiary Standards for Court Decisions

If a caregiver is charged with committing a crime, the criminal court would employ the legal standard of innocent until proven guilty beyond reasonable doubt. However, this standard is not employed in family courts for child protection cases, although the stakes for parents and children involved might be at least as high as in any criminal case (van IJzendoorn et al., 2018b). Instead, decisions are based on the balancing of probabilities and various thresholds regarding risk (Burns et al., 2016). Though proof beyond reasonable doubt may be untenable, questions arise regarding how probabilities should be balanced, when and how psychological assessment can inform this process, and how clearly the scale shall tip over for decisions such as to remove a child from its parents. In fact, scholars have emphasised that this balancing act is not sufficiently strict and that it may result in unnecessary child removals (Mnookin, 2014).

It has been reported that investments into family support are increasingly dwarfed by investments into child protection investigations and foster care services (Bilson & Martin, 2016; Granqvist, 2016). Indeed, difficulties in weighing probabilities, and misinformed applications of attachment theory for informing child protection investigations, have occasionally resulted in child removals without well-founded indications of risk for hampered development or signs of maltreatment. For instance, one study found an abundance of concerns about insecure attachment and attachment problems in child protection reports that contributed to removals of children from mothers with mild intellectual disability, and that the courts did not question the relevance of or the lack of evidence for the attachment-informed recommendations (Alexius & Hollander, 2014). Notably, there was no published research on attachment quality among children of mothers with mild intellectual disability at the time, and subsequent research has actually found a distribution of attachment classifications that aligns with what is typically found in families exposed to considerable socio-economic risk factors (Granqvist et al., 2014). Moreover, the sensitivity of these mothers has been found to be markedly heterogeneous and malleable by experience, with exposure to trauma and abuse far more important for variations in maternal sensitivity than mild intellectual deficits per se (Lindberg et al., 2017). From an attachment perspective, it is a mistake to break affectional bonds out of concerns for “insecure attachment”. Judging from their sequelae, the former (e.g. child removal) is generally a much more profound risk factor in child development than the latter. These matters are also made worse by the scarcity of experts available to assess attachment. Therefore, best-practice guidelines are rarely followed, rendering the validity of the assessments below par.

## Part II: Proposals for the use of Attachment Theory and Research in Child Protection and Custody Decisions

Having outlined problems related to applying attachment theory and research in family courts, and some likely reasons for these, we now turn to our proposed applications of attachment theory and research. We put forward three principles of attachment, based on more than half a century of research, that we believe can be used as a basis for court practitioners. We also discuss the usefulness of assessments of attachment and pertinent caregiver behaviour for guiding supportive interventions and decision-making regarding child custody and child protection. Finally, we suggest avenues for interdisciplinary collaborative research.

### 4 Three Attachment Principles Relevant to Court Practice

Attachment theory and research have great relevance for understanding factors that contribute to children’s wellbeing and socioemotional development, as well as for directing supportive interventions. Specifically, attachment theory and research is relevant for inferences about what *good-enough* care typically looks like, and how such care may be attained (van IJzendoorn et al., 2019).

#### 4.1 The Child’s Need for Familiar, Non-Abusive, and Non-Neglecting Caregivers

*The development of attachment relationships, and the benefits for psychosocial development that may stem from these relationships, depend on experiences of safe haven provision by particular, familiar, and non-abusive caregivers*.

The development and maintenance of attachment requires time and interaction. A first implication, for child custody contexts, is that limited contact with a caregiver makes it more difficult for a child to form, enhance, and maintain their expectations of that caregiver’s availability in times of need (Lamb et al., 1997). A second implication, for child protection contexts, is that almost all non-abusive and non-neglecting family-based care is likely to be better than institutional care, which is linked to highly unstable caregiving with associated developmental and mental health deficits for children (van IJzendoorn et al., 2020). In fact, these deficits are often found even where institutions provide sufficient nutritional and medical care. Such findings emphasise the importance for children to be able to develop expectations about the safe haven availability of particular, familiar, and non-abusive caregivers who are present with reliable consistency.

Other concerns must be weighed against the need for continuity with familiar caregivers. Systems for “alloparenting”, in which parental care is provided by other individuals than biological parents (e.g. in the form of foster care), can be critical in safeguarding children’s rights and interests. Thus, well-functioning emergency foster care is utterly important when children’s welfare is acutely endangered, and even temporary institutional therapeutic residential care of short duration can be required in exceptional circumstances, when therapy is urgently needed and cannot be delivered in non-residential settings (Dozier et al., 2014). Further, temporary foster care can be important when the ultimate goal is child-parent reunification, and permanent placement (including guardianship or adoption) when reunification is deemed unlikely. Yet, we hypothesise that continuing contact with caregivers is often likely to be beneficial, when safe for the child and not against the child’s welfare and explicit wish. Ultimately, attachment theory and research stress the importance of sufficiently continuous availability of familiar non-abusive, non-neglecting caregivers as a general principle (Bowlby, 1958).

### 4.2 The Value of Continuity of “Good-Enough” Care

*Expectations about safe haven availability stem from particular relationships and are not simply transferrable. Extreme caution should therefore be exercised in disrupting children’s attachment relationships*.

Safe haven provision is a property of particular, familiar relationships, and it necessitates sufficiently continuous interaction between children and caregivers. Thus, even if another caregiving environment may be better than the child’s current one on some dimension, decision-making should assign considerable weight to the value of continuity of “good-enough” care. The result is a paradox: holding families to the standard of a child’s *apparent* best interests can risk harm to his/her *true* best interests, unless those interests are taken to include the value of continuity of care. Accordingly, van IJzendoorn and colleagues (2019) have argued that good-enough care may be better suited than children’s best interests as a standard for evaluating parenting quality in court contexts. The concept of “good-enough” care, elaborated from Winnicott (1971), signifies an adequate level of meeting the child’s needs over time. This level might not be “optimal” but is sufficient to meet the child’s basic developmental needs including, but not limited to, attachment-related needs such as having a familiar safe haven. This line of reasoning is similar to that of Goldstein and colleagues (1973) who, when introducing the concept of children’s best interests, specified that what they had in mind was that the “least detrimental” option available should be selected by courts. However, this is not how the idea of “best interests” has generally been subsequently interpreted. Another take on the problem is reflected in a number of statutes in the United States, and elsewhere (e.g., Aitani, 2015; The Supreme People’s Court of the People's Republic of China, 1993), which specifically state that “continuity of care with the parents” is a factor to be considered when determining the best interests of children (USDHHS, 2016).

Of course, decisions to remove a child from his/her parents should require persuasive evidence of substantial danger to the child’s health and wellbeing, and that there are no reasonable means by which the child can be protected without removal. Consequently, family court professionals have long recognised the delicate balance between the risk of trauma from child-removal and the risk of harm from staying with the child’s original family.

Notably, the provision of safe, continuous, good-enough family-based care can be supported in various ways, and attachment researchers have developed interventions that can help parents achieve such care (Dozier et al., 2017; Steele & Steele, 2017). A number of these have been evaluated through randomised controlled trials, enabling clear conclusions about causal connections between interventions and outcomes. The results are encouraging, with numerous studies and meta-analyses demonstrating favourable intervention effects on both aspects of caregiving quality (e.g. sensitivity; Juffer et al., 2017; Moss et al., 2011) and child attachment quality (Bernard et al., 2012; Stronach et al., 2013). The interventions are also time-effective, with treatment length typically ranging between 6 and 10 sessions (Bakermans-Kranenburg et al., 2003). However, further research is needed to appraise whether attachment-based interventions can reduce the likelihood of children being placed into out-of-home care (Cyr et al., 2020).

The potential benefits of supportive interventions can be contrasted with the adverse developmental trajectories often associated with long-term out-of-home (e.g., institutional, unstable foster home) care (Berlin et al., 2011). Adoption and permanent placement in one foster home are an exception to this claim (Palacios et al., 2019), since they can, and often do, provide safe, continuous, good-enough family-based care. Other forms of long-term out-of-home care, however, have been robustly linked to a wide range of problems, including “maltreatment, drug abuse, suicidal behaviour, psychiatric morbidity, unemployment, poor school performance, teenage parenting… even after control for pertinent confounds” (van IJzendoorn et al., 2018b, p. 653).

The negative developmental trajectories associated with long-term out-of-home care should not automatically be attributed to child removal per se. Various factors that are often associated with out-of-home care may be what convey risk. For instance, adverse outcomes are likely due in part to the high risk of unstable care arrangements and placement breakdowns (e.g., the child is later returned to its original home, only to be removed once more). With variations between countries, between one fifth and two thirds of all long-term placements are broken prematurely (Konijn et al., 2018; Oosterman et al., 2007; Sallnäs et al., 2004; Wulczyn et al., 2003), with some research reporting similar findings for adoption breakdowns (Palacios et al., 2019). This comprises yet another attachment-related disruption for these already vulnerable children, with such instability leaving these children with the experience of unreliable access to a familiar safe haven. Indeed, children with multiple placement changes have been found to be at particularly high risk for adverse developmental effects, including markedly higher rates of externalising and internalising behaviour problems (Newton et al., 2000; Toussaint et al., 2018), and poorer executive functioning (Lewis et al., 2007). From an attachment perspective, courts should seek to give priority to continuity of good-enough care and be mindful of the risks entailed by temporary placements.

The value of continuity of good-enough care may also be carefully considered when the question is raised, common in many countries, of returning children from long-term stable foster care to their birth-parents, once the birth-parents’ ability to provide care is judged to have been (usually slightly) improved. Birth parents’ right to their biological children should not automatically trump children’s right to continuous, good-enough care, especially not when children have spent considerable time in stable and well-functioning foster care since a young age.

To prevent misunderstanding, we willingly acknowledge that out-of-home placements (again including temporary ones) are sometimes necessary, fully justified, and lifesaving. When placement in foster care is inevitable, it is imperative that foster parents are provided with evidence-based supportive interventions at an early stage of placement to decrease the risk of placement breakdowns. Indeed, insufficient support for foster parents likely constitutes another reason for the negative effects associated with some forms of foster care. Maltreated children can behave in ways that elicit non-nurturing and insensitive behaviours from foster caregivers, which in turn increase the risk of conflict, rejection, and placement breakdown. Available research suggests that attachment-based interventions can be effective in increasing foster parents’ sensitivity to their foster children’s signals (Bick & Dozier, 2013; Dozier et al., 2009), and facilitate positive foster-child development in a number of ways (Bernard et al., 2017; Dozier et al., 2008; Lind et al., 2017). However, it should be noted that research has thus far not been able to show that such interventions alone reduce placement disruption (Schoemaker et al., 2019).

The decision to place a child in foster care should also entail support for the child and its biological family throughout the removal process. Such interventions should start at the moment when the decision to remove the child is made, and should focus on preparing the separation, helping the child and the child’s family to understand the reasons for child removal, and reassuring them about the possibility of maintaining contact (Cassibba & Cavanna, 2018). In attachment terms, such interventions may not only reduce child and caregiver stress; maintenance of contact during placement should also provide children with the availability of a safe haven while getting to know their foster parents.

We also want to encourage attention toward structural factors that may currently lead to an increased risk of unstable placements. In some countries, children placed in out-of-home care transition to a new family if their placement status changes, such as from temporary foster care to permanent foster care, or from permanent foster care to adoption. This is because families are registered or evaluated for a certain type of care. We are also concerned that knowing that a fostered child may suddenly change status, and be placed in another family, may inadvertently decrease foster carers’ ability or willingness to invest in a child.

Finally, efforts to preserve and support families hinge on sufficient financial commitment and societal structures that support caregivers and families in a wider sense, because the ability to provide good-enough care must be seen in context. Mental health-problems, substance abuse, unemployment, lack of opportunities for education or healthcare, and lack of safe housing, make it difficult for caregivers to provide good-enough care, especially if these conditions compound. Although such complicating factors are beyond the control of family courts, they may nonetheless influence decision-making. For instance, they are relevant when assessing the likelihood that a caregiver can attain good-enough caregiving. Consequently, policy makers would do well to follow Bowlby’s (1951) admonition: “Just as children are absolutely dependent on their parents for sustenance, so in all but the most primitive communities, are parents…dependent on a greater society for economic provision. If a community values its children it must cherish their parents” (p. 84).

#### 4.3 A Network of Attachment Relationships as an Asset for Children

*Additional attachment relationships can be an asset for children. They do not typically disturb existing attachments unless they represent a source of threat or block access to existing relationships*.

The principle that a network of attachment relationships is valuable for children has implications for custody decisions: it suggests that sufficient time for the development and maintenance of attachment relationships with both caregivers is desirable, except when there is threat to the child’s welfare and safety or one of the parents wants to “opt out”. At the same time, children’s developmental age and the respective parent’s previous involvement in caring for the child must be considered. As such, attachment theory and research do not suggest any black-and-white “optimal” standard for time allocation, nor any well-specified minimum amount of time needed with each caregiver. Simply put, there is not enough empirical research for any simple answer to these questions, and the answers would again likely depend on the contexts of a given child’s development.

Theory and research on the value of a network of attachment relationships suggest that children can benefit from fairly equal time. Presuming that both parents want equal time and there are not high levels of inter-parental conflict, equal time allocation can constitute a long-term goal (Bacro et al., 2020). This conclusion is further supported by the research finding that contact frequency with the “second parent” post-divorce is a predictor of contact and relationship quality later in development (Steinbach, 2019). The principle also underlines the value of creative solutions to retain access to caregivers. A struggling teenage parent may for instance move into a foster family together with his or her child, with the possibility that both can be cared for together (Child Welfare Information Gateway, 2017).

The principle that a network of attachment relationships is valuable also has relevance to child protection. Maintaining attachment relationships with both foster and biological parents often constitutes one of the main objectives of foster care, and research has suggested that foster children can be supported to develop and maintain attachment relationships with both their biological parents and their foster parents without conflicting loyalties (Maaskant et al., 2016). Foster parents can help children explore their expectations about attachment relationships, and develop greater capacities for making use of others in times of need, thanks to experiences of caregiver availability (Cassibba & Cavanna, 2018). The principle may also inform the process of moving children from foster homes to adoptive families. This process is currently all too often abrupt, and contact with foster parents is often cut-off for a long time (Boswell & Cudmore, 2017). In contrast, transitions should allow for a substantial overlap of caregiving between foster and adoptive parents, and the maintenance of contact with foster parents, so that safe haven availability is not interrupted while children develop expectations of their adoptive parents’ availability. Except in cases of imminent risk of harm, it is difficult to imagine any circumstances in which abrupt removal of a child from one foster placement to another would be in the interests of the child. The same goes for transitions from foster carers back to birth parents.

Another implication relates to grandparents, stepparents and non-parental relationships with siblings and extended family members. In China, for example, children’s contact with grandparents can be used to decide child custody (The Supreme People’s Court of the People’s Republic of China, 1993). What matters primarily from an attachment perspective is whether a child has developed expectations of safe haven provision from these individuals, which may or may not be the case. Foster caregiver-child relationships can provide a similar emotion regulation function as parent-child relationships (Oosterman & Schuengel, 2007), and foster children have been found to be able to develop secure attachment relationships to their foster-parents within 6 to 12 months (Lang et al., 2016). However, more research is needed on how quickly an emotion regulatory function of the relationship is established, and we do not know whether pre-existing familiarity with an aunt or an uncle or a grandparent, as compared to unfamiliar foster and adoptive carers, may offer a head start for children in developing secure attachment relationships. We also know little about how and when older siblings can provide a safe haven, whether this has the same developmental benefits as safe haven provision by adults, and whether there is a price for the older sibling’s development. In a rare study of sibling attachment, conducted in Zambia where older siblings play a large role in the care of younger siblings, the majority of children had developed an attachment to their older sibling (72%), but insecure attachment was more common than secure attachment (Mooya et al., 2016).

Kinship care, and decisions to place siblings together, may certainly be justified on multiple grounds besides attachment (e.g., retaining cultural identity). It has been estimated that approximately two thirds of children in out-of-home care have siblings (Wulczyn & Zimmerman, 2005), and sibling relationships tend to carry strong emotional significance and often constitute the most enduring relationships in a person’s lifetime. Whether or not siblings have developed *attachment* relationships to one another, there are typically strong affiliative relationships between siblings, and being placed together can conceivably provide children with both a sense of continuity with their family and a sense of safety while navigating foster care. Being placed together also tends to be children’s explicit wish (Hill et al., in press).

### 5 Use of Assessments of Attachment Quality and Safe Haven Provision

Most attachment researchers agree that assessments of attachment quality can be useful for guiding supportive interventions. There are, however, different opinions among attachment researchers – the current authors included – regarding their usefulness in informing decision-making regarding child protection and child custody. Specifically, there are different opinions regarding the validity of attachment measures for such considerations. One reason for this lack of consensus likely stems from our different experiences of how attachment measures are currently used to guide assessments and decision-making in our respective countries. Such international variations likely stem from variations in infrastructure to support family courts and associated professionals, and variations in the factors contributing to misunderstandings discussed earlier. However, we all agree that assessments of the caregiver’s ability to provide a safe haven should be given greater priority than assessments of child attachment.

#### 5.1 Assessments of Attachment Quality and Child Protection

Whereas some attachment researchers have advocated for the use of attachment measures in family courts (e.g. Crittenden et al., 2013; Isaacs et al., 2009), others have cautioned against their use in this context (van IJzendoorn et al., 2018a, b). The lack of consensus depends on several factors. First and foremost, it depends on different stances regarding the current psychometric properties of attachment measures. As discussed above, attachment measures, like many other psychological instruments, currently have insufficient sensitivity and specificity for diagnostic and broader predictive purposes at the individual level. More specifically, attachment instruments, used in isolation, are not appropriate for determining the care that *individual* children receive, how *individual* children will develop, or the care arrangements that *individual* children should have (van IJzendoorn et al., 2018).

Consequently, some of us hold that family courts should be cautious in admitting as evidence any assessment of the attachment quality of a single child-caregiver relationship. In the meantime, more research is needed to improve the diagnostic properties (sensitivity and specificity) of attachment instruments and evaluate their usefulness in informing family court decision-making. Judging from the available scientific evidence, we actually do not know whether attachment measures improve family court assessment and decision-making, as compared to “assessment as usual” (which is quite variable across regions and countries). Advocacy for attachment assessments in this context (Marvin & Schutz, 2009; Spieker & Crittenden, 2018) therefore appears premature (van IJzendoorn et al., 2018a). Similarly, we currently do not know whether attachment assessments are better than assessment-as-usual in differentiating the effects of caregiving from relevant confounds such as malnutrition, developmental disorders, intellectual disability, and medication use. Incremental validity is therefore an urgent item on the research agenda.

On the other hand, family courts have to make difficult decisions, with or without the use of psychological assessments. Psychological assessment may certainly be preferable to no use of standardised assessments at all, which may increase the risk of decision-making influenced by professional biases. Some of us therefore believe that attachment assessments can be informative, if they are used responsibly. That is, various measures must be taken to maximise their validity and ensure that they are not given inappropriate weight at the expense of other considerations. More specifically, attachment measures should never be used in isolation but be part of a larger assessment battery that also includes direct assessment of caregiving behaviour. Indeed, assessment of caregiving should be the primary focus, with attachment assessments a possible complement: We should first and foremost assess the parent’s ability to understand and respond effectively to the child’s needs, to know and value the child, and to be consistently in charge in the relationship. Crucially, while the phenomenon of attachment often receives wide attention, attachment theory is at its core a theory emphasising the importance of sensitive caregiving (Bowlby & Ainsworth, 1991).

Furthermore, if attachment assessments are used to inform court deliberations, they should be employed on more than one occasion, by formally trained observers. Professionals who lack formal training should not attempt to devise their own attachment assessments or use insufficiently validated methods developed by others, and then reference children’s presumed attachment quality in recommendations to family courts. The validity of attachment measures rests on following the standardised protocols for conducting and coding them. Since both conducting and coding attachment is difficult, usage of attachment measures typically requires extensive training as well as passing a reliability test for coding. A second coder is typically also used in attachment research to ensure the reliability of the coding. As discussed above, a variety of factors can also result in children occasionally behaving in ways that are not representative (e.g. illness, recent separations, and overstress). The above standard of using attachment measures together with other measures, and examining attachment on more than one occasion, is in line with guidelines for psychological evaluation in child protection matters (e.g., American Psychiatric Association [APA], 2013).

Finally, professionals should be careful not to focus too much on the categorical classifications per se (security vs insecurity, organisation vs disorganisation), which carry similar problems to those of categorical diagnosis in psychology and psychiatry, in that they reduce nuance. For example, children assigned the same disorganised attachment classification can differ markedly from one another with respect to intensity (e.g. a score of 5 or 9 on the continuous disorganisation scale) and what sub-theme of disorganised behaviours they display (e.g. asymmetrical movements vs apprehension of the caregiver). Beyond classifications, several observational scales, using more fine-grained continuous scoring of child and caregiver behaviour, have been developed and validated, such as the Attachment Q Sort (AQS; Waters & Deane, 1985; van IJzendoorn et al., 2004), the Maternal Behavior Q Sort (MBQ; Pederson et al., 1990), and the Coding Interactive Behavior system (CIB; Feldman, 1998).

It is important to highlight that measures of attachment are designed and validated for standardised contexts. In addition, both attachment and caregiving assessments have typically been used under contexts of at most mild to moderate stress. However, assessments in forensic contexts are often conducted in highly affectively charged circumstances for caregivers and children, sometimes in the midst of child-caregiver separation. The difference in stress experienced between these contexts is an important potential confound (Smith et al., 2012b), and there is currently no research on the validity of attachment measures under such conditions. We therefore emphasise that the validity of assessments of attachment quality and caregiving behaviour is unknown under such circumstances. We realise that assessments must sometimes be carried out under stressful circumstances, and that some caregiving measures that are currently insufficiently validated for this context may represent the best available alternative. Yet, these knowledge gaps should affect the weight that is given to such evidence, and according to some of us, non-standardised observations by experienced professionals offer at least as much credible knowledge and perhaps more.

Assessment of a caregiver’s capacity for enhanced caregiving may constitute a solution to this dilemma. More specifically, the caregiver’s *potential* to provide good-enough care represents the outcome that is sought, and assessments may thus evaluate whether a caregiver is likely able to improve his or her caregiving to such a level. Families may for instance receive an intervention aimed at avoiding harsh discipline and promoting consistent, sensitive caregiving, and caregiving and child behaviour may be assessed before and after intervention as an indication of how the caregiver is likely to respond to future support. Importantly, such interventions can be brief, making the approach feasible within a short time-frame. Initial findings from this “capacity to change” approach have indicated better predictive validity for parent and child outcomes than assessment-as-usual (Cyr et al., 2012). Notably, however, the effectiveness of such interventions may depend on contextual factors that influence the caregiver’s receptiveness. For instance, caregivers who experience very high levels of stress, due to an acute risk of losing custody of their children, may not be able to benefit. Indeed, a recent randomised control trial of families studied at the end of the forensic process, who were given such an intervention as a last chance after a long trajectory of home-based support, reported no difference in prediction compared to assessment-as-usual (Van der Asdonk et al., in press).

Although further evidence is needed, interventions should be prioritised early in the investigation process and given in a compassionate, supportive manner rather than as a “last straw”. Furthermore, it should be acknowledged that a caregiver’s response to a particular intervention might also be a function of both its suitability and the quality of its delivery: if a caregiver does not respond to one evidence-based intervention, he/she may respond to another one, bearing in mind the child's age, need for permanency, and capacity to wait.

#### 5.2 Assessments of Attachment Quality and Child Custody

Some scholars have suggested that assessments of attachment may aid in deciding about child custody and time allocation, or observed the use of these assessments for such a purpose by courts (e.g. Aitani, 2015; Kohm, 2007). This advocacy is ill-advised because it is currently unknown whether, or in which ways, children benefit from more time with a parent with whom they are secure than with a parent with whom they are insecure. Moreover, considerably depriving a child of time with a parent is in itself a risk factor for insecurity and disorganisation in that relationship (Hazen et al., 2015; Umemura & Jacobvitz, 2014). Returning to the distinction between attachment quality and overall relationship quality, insecurity does not mean that a child does not benefit from the relationship with a parent. Insecurity has probabilistic long-term disadvantages with regard to some aspects of child development; but it is not pathological. Overemphasis on secure attachment may therefore deprive children of time with caregivers from whom they benefit in other areas. Finally, we do not know to what extent assessments of attachment are valid during custody disputes, when parents and children may appear more anxious due to the ongoing conflict and its ramifications.

In custody disputes, family courts frequently work with adults who are hurt, focused on their own needs, and sometimes motivated to inflict pain on the other parent. Children can get caught in the middle of such acrimonious conflicts, and adversarial processes may further inflame conflicts and make things worse. Indeed, it has long been held that one of the most harmful things about divorce for children may be the inter-parental conflict witnessed before, during, and after divorce (Amato & Keith, 1991; Kalmijn, 2016). Indeed, chronic marital conflict has been linked to increased risk of disorganised child attachment (Owen & Cox, 1997). Courts can fill an important role in shaping the dynamic between caregivers and the legal process should encourage caregivers to work out their own resolutions without the need for formal adjudication (Mnookin, 2014; Pruett et al., 2016). Clarity in decision-making regarding custody and time allocation, including how factors relating to child attachment are evaluated, may therefore improve caregivers’ capacity for cooperation over conflict and affect whether or not caregivers fight for sole custody. For instance, if it is made clear that courts draw upon the attachment principles elaborated above, including the importance of a network of attachment relationships and continuous contact with each attachment figure, this may influence caregivers’ awareness of the importance of the other caregiver for the child. This may, in turn, improve caregivers’ motivation for cooperation and prevent them from fighting for sole custody. Also, knowing that courts do not base decision-making regarding custody and time allocation on the child’s purported attachment quality to each caregiver may reduce fights over sole custody based on such references.

If post-divorce conflict cannot be resolved, and caregivers cannot find a good-enough way of cooperating, sole custody may be inevitable as a last resort. However, decision-making regarding time-allocation can still ensure that the child gets enough time with both parents for development and maintenance of attachment relationships. Following the notion of “cooperative parenting” (Boyan & Termini, 1999), some countries such as Sweden take the parents’ ability to cooperate around their child(ren) into consideration in custody-related decision-making. If forced to make a decision on child custody, an emphasis is placed on the extent to which the respective parents have facilitated or hindered the child’s contact with the other parent. For instance, if one parent has obstructed the child’s contact with and transitions to the other parent, whereas the other parent has facilitated contact and transitions, custody is often awarded to the parent who has demonstrated an ability to act in the child’s interests. Of course, this principle must also be communicated and implemented with judgement, to prevent that divorced caregivers refrain from initiating mutual discussions about caregiving practices due to fear of being perceived as non-cooperative.

#### 5.3 Assessment of Attachment, Safe haven Provision, and Identifying and Targeting Future Support

If insecure attachment appears to be present in a child’s relationship with a caregiver, it should not be ignored because it may inform supportive interventions. However, we regard assessments of a caregiver’s capacity to provide a safe haven for the child when alarmed as more valuable for targeting supportive interventions than is information about the child’s attachment classification per se, especially when provision of more effective caregiving is the key concern. Such safe haven assessments have been developed for children of various ages and may be used in naturalistic settings (Farnfield & Holmes, 2014; Madigan, 2019), though their application in practice has yet to be adequately validated. Obstacles to safe haven provision are also of special importance when identifying and targeting support (e.g. caregiving interventions) for families in child protection and custody contexts. Furthermore, the problems with the diagnostic precision of the assessments are considerably less serious when used to target supportive interventions than when the purpose is to decide whether or not to place a child in out-of-home care (for a similar argument, see Faigman et al., 2014). Though caregivers may still be afraid that their child may eventually be placed in out-of-home custody, clarification that the purpose is to direct supportive work may help in achieving the contexts of mild/moderate challenge or naturalistic settings for which the measures were developed and validated.

To illustrate, indications of an avoidant attachment relationship may offer a window in to the child’s probable expectations about that relationship. To learn that there is an elevated probability that a child expects that a caregiver will reject her when she is upset (i.e., insecure-avoidant attachment) can be helpful for deciding priorities in supportive intervention with the family, whether it is in the context of biological parents or foster parents (Brumariu et al., 2018; Green et al., 2000). Even more helpful would be an assessment of caregiving in which the caregiver’s safe haven provision is found to be limited by rejection of the child’s attempts to gain availability. This is more direct, and therefore more relevant, than an assessment of the child’s attachment quality.

With such information, professionals may support the family in identifying when the child is upset, even if she does not overtly show this, and how to respond appropriately. Moreover, and of particular importance in foster home contexts, explaining to caregivers how the child’s caregiving history may predispose her to certain behaviours may help caregivers regulate feelings of being rejected when the child does not seek support, and to stay available to the child (Stovall & Dozier, 2000). Conversely, to learn that there is an elevated probability that a child expects adults to be primarily available if the child shows high levels of distress and seeks high degrees of availability (i.e. insecure-resistant attachment) suggests a different track. Again, however, an assessment of actual caregiving in which this dynamic is observed directly would provide stronger, less inferential information. Professionals may then support the family in responding consistently and conveying that availability is not conditional on displays of distress. Assessment of caregivers’ attachment representations may also be useful in targeting support. For instance, secure attachment representations in adoptive parents of institutionalised children have been linked to an increased likelihood of secure child attachment (Barone et al., 2017).

### 6 Future Research

No doubt, there are areas for future research that can most readily be identified by practitioners in this area. As a community, we are eager to engage in dialogue with practitioners, and we look for opportunities for collaborative, co-constructed research initiatives. For our part, we perceive particular need for collaborative research in the following areas. The collaborative research that we envision here can ideally help to close the research-practice gap and build infrastructures to support accurate knowledge transfer (Nicolini et al., 2012).

#### 6.1 Court Decisions and Subsequent Child Attachment Quality

There is good reason to believe that decisions regarding child custody and child protection have an impact upon child attachment quality, but there is currently very little empirical knowledge. Regarding custody cases, research is needed to address whether differences in time allocation are associated with differences in child attachment. For instance, is joint legal custody and equal time allocation associated with higher rates of secure attachment than sole custody and unequal time allocation? Such research should also examine factors that may influence the association between time allocation and child attachment, such as inter-parental conflict.

Regarding child protection, research should examine whether, and under which circumstances, child removals are associated with higher levels of attachment security than if children remain within their original families. Although several studies have examined children’s attachment relationships with their foster parents (e.g., Gabler et al., 2014; Van der Dries et al., 2009), research could compare child attachment to foster parents with attachment to birth parents who are provided with a supportive intervention. The potential relevance of the type of maltreatment and developmental timing should be examined. Similarly, research is needed to examine whether, and under which circumstances, returning children to their birth parents after out-of-home care is associated with higher rates of attachment security than if children remain in foster care. Answers to these questions are of crucial importance to the courts’ aim of supporting children’s best interests and/or ensuring provision of good enough care; even if attachment security is only one part of a positive child-caregiver relationship, it is an important part.

#### 6.2 The Three Attachment Principles and Court Practice

We have emphasised children’s need for familiar non-abusive and non-neglecting caregivers, continuity of good-enough care, and a network of attachment relationships as fundamental principles of attachment theory and developmental science. Can knowledge about these principles improve court practice and social work assessment regarding key metrics such as quality of care, continuity of good-enough care, subsequent abuse or neglect, and child wellbeing? In addition to addressing these key matters, we call for research to address the following related, but more specific, questions:

- Are attachment measures suitable for guiding supportive interventions, and is inclusion of attachment assessments better than assessment as usual? For instance, are supportive interventions guided by attachment assessment better than interventions not guided by such assessments? Similarly, are interventions guided by assessments of caregiving and attachment more effective at improving caregiving quality and child development than interventions not guided by such assessments?
- Are assessments of parental capacity to change (response to intervention) following brief interventions sufficiently reliable and valid? How does parental fear and despair affect the validity of such assessments?
- Do multiple assessments of caregiving in forensic contexts have higher predictive value than one assessment? If so, are multiple assessments more cost-effective than single assessments? Assessments of caregiving (and attachment) are costly. However, ineffective interventions and placements in foster care are also costly. A developmentally-informed health-economic evaluation of the potential economic benefits of conducting multiple assessments would be important.
- How does switching between two parental homes after parental separation during the first years of life influence attachment development? Does development of attachment and attachment quality depend on contact frequency, and/or overnights with a caregiver? Do arrangements where the child stays in one familiar home and the separated parents rotate to be there with the child facilitate development and maintenance of secure attachment relationships? Moreover, are these associations moderated by parental conflict and cooperation, and children’s developmental age? How can this knowledge be used in court decisions?
- Is provision of safe haven by an older sibling, in the context of fostering/adoption together, associated with harm and/or benefit to the older sibling over time, and is it beneficial and/or harmful to the younger sibling over time?
- Do familiar and/or kinship carers have a head-start for children’s development of secure attachment and other indices of healthy development (including protecting cultural identity), compared to unfamiliar foster and adoptive carers?

Answering these and other pertinent questions, identified by social work and family law practitioners and academics, would likely be facilitated by greater collaboration across our respective disciplines. Co-developed research questions and co-construction of standards for appropriate applications of research findings have the potential to greatly benefit both research and practice (Madigan, 2019; van IJzendoorn, 2019). In brief, we invite dialogue and the initiation of co-constructed efforts.

## Conclusion

Family courts are in a very challenging position, having to make difficult, life changing, and potentially life-saving, decisions. Such decisions demand that probabilities are weighed concerning future child development. We have argued for the relevance of attachment theory and research for supporting children and their caregivers. More specifically, we emphasised three foundational attachment principles that may be used to guide court deliberation: the child’s need for familiar, non-abusive caregivers; the value of continuity of good-enough care; and the benefits of networks of attachment relationships. In addition, we highlighted the promise of both caregiving and attachment-based assessment for informing supportive interventions. Trials have also demonstrated that attachment relationships are responsive to evidence-based caregiving interventions.

It is imperative to provide families with support to facilitate good-enough care, and not threaten continuity of care without the most serious of justifications. Furthermore, we argued that although child removal is sometimes warranted, there are risks associated with breaking established attachment bonds, and it often leads to unstable out-of-home care arrangements with adverse consequences for the child’s development. Therefore, when removal is inevitable, which of course it sometimes is, it is imperative to achieve stable placements marked by good-enough care. When removal is not inevitable, children’s best interests can be supported by settling for and helping caregivers to provide continuous good-enough care.

We highlighted that measures to assess attachment quality, which were developed for research at the group-level, have limited sensitivity and specificity at the level of *individual* children and caregivers. Many attachment researchers therefore believe that attachment classifications should not be used to guide decision-making regarding child custody and child protection, which should instead focus on caregiving behaviour. However, other attachment researchers believe that attachment assessments can be useful in this context, emphasising – among other important considerations – that such observations must then be part of a larger assessment battery that also includes observations of caregiving behaviour.

Finally, we suggested avenues for collaborative work between attachment researchers and family court academics and practitioners. Through interdisciplinary collaboration, we look forward to accelerating work in this exceedingly important area of applied science.

## Footnotes

1 Not all countries have family courts but instead use other institutions (e.g., juvenile courts) to decide in the cases relevant here. Nonetheless, throughout this paper we use “family courts” for lack of a more suitable generic term, to refer to the societal institutions responsible for legal decisions regarding child protection and/or child custody.

2 According to article 12 of the UNCRC (1989), the view of the child should be given due weight. In the context of parental divorce and custody assessments, one way to identify a child’s view is, of course, to ask the child to state it, and this is often done when deemed developmentally appropriate (e.g. from around child age 10 – 12). However, this is difficult with younger children, who are the main focus of the current paper, in which case observing how they respond to their caregivers can represent a way to gain insight.

## References

[R1] Abraham E, Feldman R (2018). The neurobiology of human allomaternal care; implications for fathering, coparenting, and children’s social development. Physiology & Behavior.

[R2] Ainsworth MDS, Bell SM, Stayton DJ, Richards JM (1974). The integration of a child into a social world.

[R3] Ainsworth MDS, Blehar MC, Waters E, Wall SN ([1978] 2015). Patterns of attachment: A psychological study of the strange situation.

[R4] Aitani N (2015). A new psychological method for determining child custody. Journal of Human Environmental Studies.

[R5] Alexius K, Hollander A (2014). Care assessments concerning involuntary removal of children from intellectually disabled parents. Journal of Social Welfare and Family Law.

[R6] Allen B (2018). Misperceptions of reactive attachment disorders persist: Poor methods and unsupported conclusions. Research in Developmental Disabilities.

[R7] Allen B, Schuengel C (2020). Attachment disorders diagnosed by community practitioners: a replication and extension. Child and Adolescent Mental Health.

[R8] Amato PR, Keith B (1991). Parental divorce and the well-being of children: A meta-analysis. Psychological Bulletin.

[R9] American Psychological Association (2013). Guidelines for psychological evaluations in child protection matters. The American Psychologist.

[R10] Artis JE (2004). Judging the best interests of the child: Judges' accounts of the tender years doctrine. Law & Society Review.

[R11] Bachmann CJ, Beecham J, O'Connor TG, Scott A, Briskman J, Scott S (2019). The cost of love: financial consequences of insecure attachment in antisocial youth. Journal of Child Psychology and Psychiatry.

[R12] Bacro F, Forslund T, Granqvist P, Forslund T, Duschinsky R (2020). Attachment: A reader.

[R13] Bacro F, Macario de Medeiros JM Externalizing behavior and attachment disorganization in children of different-sex separated parents: The protective role of joint physical custody. Scandinavian Journal of Psychology.

[R14] Bakermans-Kranenburg MJ, van Ijzendoorn MH, Juffer F (2003). Less is more: Meta-analyses of sensitivity and attachment interventions in early childhood. Psychological Bulletin.

[R15] Barnombudsmannen (2007). Klara, färdiga, Gå!.

[R16] Barone L, Lionetti F, Green J (2017). A matter of attachment? How adoptive parents foster post-institutionalized children’s social and emotional adjustment. Attachment & Human Development.

[R17] Barone L, Ozturk Y, Lionetti F (2019). The key role of positive parenting and children’s temperament in post-institutionalized children’s socio-emotional adjustment after adoption placement. A RCT study. Social Development.

[R18] Barudy J, Dantagnan M (2010). Los desafíos invisibles de ser madre y padre: Manual de evaluación de las competencias y la resiliencia parental.

[R19] Belsky J (1997). Attachment, mating, and parenting: An evolutionary interpretation. Human Nature.

[R20] Belsky J, Bakermans-Kranenburg MJ, Van IJzendoorn MH (2007). For better and for worse: Differential susceptibility to environmental influences. Current Directions in Psychological Science.

[R21] Belsky J, Rovine M (1987). Temperament and attachment security in the strange situation: An empirical rapprochement. Child Development.

[R22] Berlin M, Vinnerljung B, Hjern A (2011). School performance in primary school and psychosocial problems in young adulthood among care leavers from long term foster care. Children and Youth Services Review.

[R23] Bernard K, Dozier M, Bick J, Lewis-Morrarty E, Lindhiem O, Carlson E (2012). Enhancing attachment organization among maltreated children: Results of a randomized control trial. Child Development.

[R24] Bernard K, Lee AH, Dozier M (2017). Effects of the ABC intervention on foster children’s receptive vocabulary: Follow-up results from a randomized clinical trial. Child Maltreatment.

[R25] Bernier A, Meins E (2008). A Threshold Approach to Understanding the Origins of Attachment Disorganization. Developmental Psychology.

[R26] Bick J, Dozier M (2013). The effectiveness of an attachment-based intervention in promoting foster mothers’ sensitivity toward foster infants. Infant Mental Health Journal.

[R27] Bifulco A, Jacobs C, Bunn A, Thomas G, Irving K (2008). The attachment style interview (ASI): A support-based adult assessment tool for adoption and fostering practice. Adoption & Fostering.

[R28] Bilson A, Martin KE (2017). Referrals and child protection in England: One in five children referred to children’s services and one in nineteen investigated before the age of five. British Journal of Social Work.

[R29] Boldt LJ, Kochanska G, Yoon JE, Nordling JK (2014). Children’s attachment to both parents from toddler age to middle childhood: Links to adaptive and maladaptive outcomes. Attachment & Human Development.

[R30] Boswell S, Cudmore L (2017). Understanding the ‘blind spot’when children move from foster care into adoption. Journal of Child Psychotherapy.

[R31] Bowlby J (1951). Maternal care and mental health.

[R32] Bowlby J (1958). Separation of mother and child. Letter to the editor. The Lancet.

[R33] Bowlby J (1969/1982). Attachment and loss: Attachment.

[R34] Bowlby J (1973). Attachment and loss: Separation.

[R35] Bowlby J (1980). Attachment and loss: Loss.

[R36] Bowlby (1984). personal communication.

[R37] Bowlby J, Robertson J, Rosenbluth D (1952). A two-year-old goes to hospital. The Psychoanalytic Study of the Child.

[R38] Boyan S, Termini AM (1999). Cooperative parenting and divorce: Shielding your child from conflict – a parent guide to effective co-parenting.

[R39] Brown GL, Schoppe-Sullivan SJ, Mangelsdorf SC, Neff C (2010). Observed and reported supportive coparenting as predictors of infant–mother and infant–father attachment security. Early Child Development and Care.

[R40] Brown R, Ward H (2013). Decision-making within a child’s timeframe: An overview of current research evidence for family justice professionals concerning child development and the impact of maltreatment.

[R41] Brumariu LE, Giuseppone KR, Kerns KA, Van de Walle M, Bureau J-F, Bosmans G, Lyons-Ruth K (2018). Middle childhood attachment strategies: Validation of an observational measure. Attachment & Human Development.

[R42] Bullens RAR, Schuengel C, Slot NW, Bullens RAR (2003). Gehechtheid en kinderbescherming.

[R43] Bunnvik G (2016). Lurades till möte – miste barnen. Jnytt.

[R44] Burns K, Pösö T, Skivenes M (2016). Child welfare removals by the state: A cross-country analysis of decision-making systems.

[R45] Byrne JG, O’Connor TG, Marvin RS, Whelan WF (2005). Practitioner review: The contribution of attachment theory to child custody assessments. Journal of Child Psychology and Psychiatry.

[R46] Carlson V, Cicchetti D, Barnett D, Braunwald K (1989). Disorganized/disoriented attachment relationships in maltreated infants. Developmental Psychology.

[R47] Cassibba R, Cavanna D (2018). L’affidamento familiare tra teoria e realtà: opportunità, incongruenze e contraddizioni [The foster care between theory and reality: opportunities, inconsistencies and contradictions]. Psicologia Clinica dello Sviluppo.

[R48] Chaffin M, Hanson R, Saunders BE, Nichols T, Barnett D, Zeanah C, LeTourneau E (2006). Report of the APSAC task force on attachment therapy, reactive attachment disorder, and attachment problems. Child Maltreatment.

[R49] Cheadle JE, Amato PR, King V (2010). Patterns of nonresident father contact. Demography.

[R50] Child Welfare Information Gateway (2017). Concurrent planning for permanency for children.

[R51] Cowan PA, Cowan CP (2019). Introduction: bringing dads back into the family. Attachment & Human Development.

[R52] Crittenden PM, Baim C, Dixon L, Perkins DF, Hamilton-Giachritis C, Craig LA (2017). What works in child protection: An evidenced based approach to assessment and intervention in care proceedings.

[R53] Crittenden PM, Farnfield S, Landini A, Grey B (2013). Assessing attachment for family court decision making. Journal of Forensic Practice.

[R54] Cyr C, Dubois-Comtois MG, Poulin C, Pascuzzo K, Losier V, Dumais M, St-Laurent D, Moss E, Muela A (2012). Child abuse and neglect: A multidimensional approach.

[R55] Cyr C, Euser EM, Bakermans-Kranenburg M, van IJzendoorn M (2010). Attachment security and disorganization in maltreating and high-risk families: A series of meta-analyses. Development and Psychopathology.

[R56] Cyr C, Dubois-Comtois K, Paquette D, Lopez L, Bifgras M (2020). An attachment-based parental capacity assessment with a focus on capacity to change to orient decision-making in child protection cases.

[R57] Dagan O, Sagi-Schwartz A (2018). Early attachment network to mother and father: An unsettled issue. Child Development Perspectives.

[R58] Departamento de Protección de Derechos Servicio Nacional de Menores (2019). Orientaciones técnicas: Línea de acción diagnóstico modalidad diagnóstico ambulatorio, DAM.

[R59] Department of Health (2000). Assessing children in need and their families: Practice guidance.

[R60] Department for Education (2018). Children in need of help and protection: Call for evidence.

[R61] De Wolff MS, Van Ijzendoorn MH (1997). Sensitivity and attachment: A meta-analysis on parental antecedents of infant attachment. Child Development.

[R62] Dozier M, Bernard K, Roben C, Steele H, Steele M (2017). Handbook of attachment-based interventions.

[R63] Dozier M, Kaufman J, Kobak R, O'Connor TG, Sagi-Schwartz A, Scott S, Schauffer C, Smetana J, van IJzendoorn MH, Zeanah CH (2014). Consensus statement on group care for children and adolescents: A statement of policy of the American Orthopsychiatric Association. American Journal of Orthopsychiatry.

[R64] Dozier M, Lindhiem O, Lewis E, Bernard K, Peloso E (2009). Effects of a foster parent training program on young children’s attachment behaviors: Preliminary evidence from a randomized clinical trial. Child and Adolescent Social Work Journal.

[R65] Dozier M, Peloso E, Lewis E, Laurenceau J, Levine S (2008). Effects of an attachment-based intervention of the cortisol production of infants and toddlers in foster care. Development and Psychopathology.

[R66] Duschinsky R (2020). Cornerstones of attachment research.

[R67] Duschinsky R, Van IJzendoorn M, Foster S, Reijman S, Lionetti F (2019). Attachment histories and futures: Reply to Vicedo’s ‘Putting attachment in its place’. European Journal of Developmental Psychology.

[R68] Egeland B, Jacobvitz D, Sroufe LA (1988). Breaking the cycle of abuse. Child Development.

[R69] Emery RE, Holtzworth-Munroe A, Johnston JR, Pedro-Carroll JL, Pruett MK, Saini M, Sandler I (2016). “Bending” evidence for a cause: Scholar-advocacy bias in family law. Family Court Review.

[R70] Emery RE, Otto RK, O’donohue WT (2005). A critical assessment of child custody evaluations: Limited science and a flawed system. Psychological Science in the Public Interest.

[R71] Fabricius WV, Suh GW (2017). Should infants and toddlers have frequent overnight parenting time with fathers? The policy debate and new data. Psychology, Public Policy, and Law.

[R72] Facompré CR, Bernard K, Waters TEA (2018). Effectiveness of interventions in preventing disorganized attachment: A meta-analysis. Development and Psychopathology.

[R73] Faigman DL, Monahan J, Slobogin C (2014). Group to individual (G2i) inference in scientific expert testimony. The University of Chicago Law Review.

[R74] Faigman DL, Slobogin C, Monahan J (2016). Gatekeeping science: using the structure of scientific research to distinguish between admissibility and weight in expert testimony. Northwestern University Law Review.

[R75] Farnfield S, Holmes P (2014). The Routledge handbook of attachment: Assessment.

[R76] Fearon RP, Bakermans-Kranenburg MJ, Van IJzendoorn MH, Lapsley AM, Roisman GI (2010). The significance of insecure attachment and disorganization in the development of children’s externalizing behavior: A meta-analytic study. Child Development.

[R77] Fearon RMP, Belsky J, Cassidy J, Shaver P (2016). Handbook of attachment: Theory, research, and clinical applications.

[R78] Feldman R (1998). Coding interactive behavior manual.

[R79] Feldman R, Bamberger E, Kanat-Maymon Y (2013). Parent-specific reciprocity from infancy to adolescence shapes children's social competence and dialogical skills. Attachment & Human Development.

[R80] Feldman R, Braun K, Champagne FA (2019). The neural mechanisms and consequences of paternal caregiving. Nature Reviews Neuroscience.

[R81] Feldman R, Masalha S (2007). The role of culture in moderating the links between early ecological risk and young children's adaptation. Development and Psychopathology.

[R82] Font SA, Gershoff ET (2020). Foster care and best interests of the child: Integrating research, policy, and practice.

[R83] Forslund T, Peltola MJ, Brocki KC (2019). Disorganized attachment representations, externalizing behavior problems, and socioemotional competences in early school-age. Attachment & Human Development.

[R84] Funder DC, Ozer DJ (2019). Evaluating effect size in psychological research: Sense and nonsense. Advances in Methods and Practices in Psychological Science.

[R85] Gabler S, Bovenschen I, Lang K, Zimmermann J, Nowacki K, Kliewer J, Spangler G (2014). Foster children’s attachment security and behavior problems in the first six months of placement: Associations with foster parents’ stress and sensitivity. Attachment & Human Development.

[R86] Garber BD (2009). Attachment methodology in custody evaluation: Four hurdles standing between developmental theory and forensic application. Journal of Child Custody.

[R87] Gauthier Y, Fortin G, Jéliu G (2004). Clinical application of attachment theory in permanency planning for children in foster care: The importance of continuity of care. Infant Mental Health Journal.

[R88] George C, Solomon J (2008). The caregiving system: A behavioral systems approach to parenting. Handbook of attachment: Theory, research, and clinical applications.

[R89] GM v (2018). Carmarthenshire County Council. EWFC.

[R90] Goldstein J, Freud A, Solnit AJ (1973). Beyond the best interests of the child.

[R91] Granqvist P (2016). Observations of disorganized behaviour yield no magic wand: Response to Shemmings. Attachment & Human Development.

[R92] Granqvist P, Forslund T, Fransson M, Springer L, Lindberg L (2014). Mothers with intellectual disability, their experiences of maltreatment, and their children’s attachment representations: A small-group matched comparison study. Attachment & Human Development.

[R93] Granqvist P, Hesse E, Fransson M, Main M, Hagekull B, Bohlin G (2016). Prior participation in the strange situation and overstress jointly facilitate disorganized behaviours: Implications for theory, research and practice. Attachment & Human Development.

[R94] Granqvist P, Sroufe LA, Dozier M, Hesse E, Steele M, van IJzendoorn M, Solomon J, Schuengel C, Fearon P, Bakermans-Kranenburg M, Steele H (2017). Disorganized attachment in infancy: A review of the phenomenon and its implications for clinicians and policy-makers. Attachment & Human Development.

[R95] Green J, Stanley C, Smith V, Goldwyn R (2000). A new method of evaluating attachment representations in young school-age children: The Manchester Child Attachment Story Task. Attachment & Human Development.

[R96] Groh AM, Fearon RP, van IJzendoorn MH, Bakermans-Kranenburg MJ, Roisman GI (2017). Attachment in the early life course: Meta-analytic evidence for its role in socioemotional development. Child Development Perspectives.

[R97] Groh AM, Narayan AJ, Bakermans-Kranenburg MJ, Roisman GI, Vaughn BE, Fearon RP, van IJzendoorn MH (2017). Attachment and temperament in the early life course: A meta-analytic review. Child Development.

[R98] Grossmann K, Sagi-Schwartz A (2013). Attachment and Divorce: Facts, Myths and Dilemmas in Custody Disputes.

[R99] Grossmann K, Grossmann KE, Kindler H, Zimmermann P, Cassidy J, Shaver PR (2008). Handbook of attachment: Theory, research, and clinical applications.

[R100] Hacker D, Halperin Kaddari R (2013). The ruling rules in custody disputers – On the dangers of the parental sameness illusion in a gendered reality. Mishpat and Mimshal.

[R101] Harmer AL, Goodman-Delahunty J (2014). Practitioners’ opinions of best interests of the child in Australian legislation. Psychiatry, Psychology and Law.

[R102] Hazen NL, Allen SD, Christopher CH, Umemura T, Jacobvitz DB (2015). Very extensive nonmaternal care predicts mother-infant attachment disorganization: Convergent evidence from two samples. Development & Psychopathology.

[R103] Hill L, Gilligan R, Connelly G How did kinship care emerge as a significant form of placement for children in care? A comparative study of the experience in Ireland and Scotland. Children and Youth Services Review.

[R104] Hrdy SB (2011). Mothers and others.

[R105] Huntington C (2018). The empirical turn in family law. Columbia Law Review.

[R106] Isaacs MB, George C, Marvin RS (2009). Utilizing attachment measures in child custody evaluations: Incremental validity. Journal of Child Custody.

[R107] Jacobs S (1997). The hidden gender bias behind the best interest of the child standard in custody decisions. Georgia State University Law Review.

[R108] Jacobvitz D, Leon K, Hazen N (2006). Does expectant mothers' unresolved trauma predict frightening/frightened maternal behavior? Risk and protective factors. Development and Psychopathology.

[R109] Joels T, Sagi-Schwartz A (2012). "Mom, dad, and what about me, I need you both”: Facts, myths and hopes in custody disputes. Din Udvarim (Haifa Law Review).

[R110] Juffer F, Bakermans-Kranenburg MJ, van IJzendoorn MH (2017). Pairing attachment theory and social learning theory in video-feedback intervention to promote positive parenting. Current Opinion in Psychology.

[R111] Kelly JB, Lamb ME (2000). Using child development research to make appropriate custody and access decisions for young children. Family and Conciliation Court Review.

[R112] Kelly JB, Lamb ME (2003). Developmental issues in relocation cases involving young children: When, whether, and how?. Journal of Family Psychology.

[R113] Kohm LM (2007). Tracing the foundations of the best interests of the child standard in American jurisprudence. Journal of Law & Family Studies.

[R114] Konijn C, Admiraal S, Baart S, van Rooij F, Stams G-J, Colonnesi C, Lindauer R, Assink M (2018). Foster care placement instability: A meta-analytic review. Children and Youth Services Review.

[R115] Kvarven A, Strømland E, Johannesson M (2020). Comparing meta-analyses and preregistered multiple-laboratory replication projects. Nature Human Behaviour.

[R116] Lamb ME (2012). A wasted opportunity to engage with the literature on the implications of attachment research for family court professionals. Family Court Review.

[R117] Lamb M (2018). Does shared parenting by separated parents affect the adjustment of young children?. Journal of Child Custody.

[R118] Lamb ME, Sternberg KJ, Thompson RA (1997). The effects of divorce and custody arrangements on children’s behavior, development, and adjustment. Family and Conciliation Courts Review.

[R119] Lang K, Bovenschen I, Gabler S, Zimmermann J, Nowacki K, Kliewer J, Spangler G (2016). Foster children’s attachment security in the first year after placement: A longitudinal study of predictors. Early Childhood Research Quarterly.

[R120] Lee SM, Borelli JL, West JL (2011). Children’s attachment relationships: Can attachment data be used in child custody evaluations?. Journal of Child Custody.

[R121] Lewis EE, Dozier M, Ackerman J, Sepulveda-Kozakowski S (2007). The effect of placement instability on adopted children’s inhibitory control abilities and oppositional behavior. Developmental Psychology.

[R122] Lind T, Raby KL, Caron EB, Roben CKP, Dozier M (2017). Enhancing executive functioning among toddlers in foster care with an attachment-based intervention. Development and Psychopathology.

[R123] Lindberg L, Fransson M, Forslund T, Springer L, Granqvist P (2017). Maternal sensitivity in mothers with mild intellectual disabilities is related to experiences of maltreatment and predictive of child attachment: A matched-comparison study. Journal of Applied Research in Intellectual Disabilities.

[R124] Lionetti F, Pastore M, Barone L (2015). Attachment in institutionalized children: A review and meta-analysis. Child Abuse & Neglect.

[R125] Lucassen N, Tharner A, Van Ijzendoorn MH, Bakermans-Kranenburg MJ, Volling BL, Verhulst FC, Tiemeier H (2011). The association between paternal sensitivity and infant-father attachment security: A meta-analysis of three decades of research. Journal of Family Psychology.

[R126] Lux U, Walper S (2019). A systemic perspective on children’s emotional insecurity in relation to father: Links to parenting, interparental conflict and children’s social well-being. Attachment & Human Development.

[R127] Lyons-Ruth K, Jacobvitz D, Cassidy J, Shaver PR (2016). Handbook of attachment: Theory, research and clinical applications.

[R128] Maaskant AM, van Rooij FB, Bos HMW, Hermanns JMA (2016). The wellbeing of foster children and their relationship with foster parents and biological parents: a child’s perspective. Journal of Social Work Practice.

[R129] Madigan S (2019). Beyond the academic silo: Collaboration and community partnerships in attachment research.

[R130] Madigan S, Bakermans-Kranenburg MJ, Van Ijzendoorn MH, Moran G, Pederson DR, Benoit D (2006). Unresolved states of mind, anomalous parental behavior, and disorganized attachment: A review and meta-analysis of a transmission gap. Attachment & Human Development.

[R131] Main M (1990). Cross-cultural studies of attachment organization: Recent studies, changing methodologies, and the concept of conditional strategies. Human Development.

[R132] Main M, Hesse E, Greenberg MT, Cicchetti D, Cummings EM (1990). The John D and Catherine T MacArthur Foundation series on mental health and development Attachment in the preschool years: Theory, research, and intervention.

[R133] Main M, Hesse E, Hesse S (2011). Attachment theory and research: Overview with suggested applications to child custody. Family Court Review.

[R134] Main M, Solomon J, Brazelton TB, Yogman MW (1986). Affective development in infancy.

[R135] Marvin RS, Schutz BM (2009). One component of an evidence-based approach to the use of attachment research in child custody evaluations. Journal of Child Custody.

[R136] McIntosh J (2011). Guest editor’s introduction to special issue on attachment theory, separation and divorce: Forging coherent understandings for family law. Family Court Review.

[R137] McIntosh JE, Smyth BM, Kelaher MA (2015). Responding to concerns about a study of infant overnight care postseparation, with comments on consensus: Reply to Warshak (2014). Psychology, Public Policy, and Law.

[R138] McIntosh JE, Smyth BM, Kelaher M (2013). Overnight care patterns following parental separation: Associations with emotion regulation in infants and young children. Journal of Family Studies.

[R139] Mesman J, Van IJzendoorn MH, Sagi-Schwartz A, Cassidy J, Shaver P (2016). Handbook of attachment: Theory, research, and clinical applications.

[R140] Mercer J (2019). Conventional and unconventional perspectives on attachment and attachment problems: Comparisons and implications, 2006–2016. Child and Adolescent Social Work Journal.

[R141] Mnookin RH (1975). Child-custody adjudication: Judicial functions in the face of indeterminacy. Law & Contemporary Problems.

[R142] Mnookin R (2014). Child custody revisited. Law & Contemporary Problems.

[R143] Mooya H, Sichimba F, Bakermans-Kranenburg M (2016). Infant–mother and infant–sibling attachment in Zambia. Attachment & Human Development.

[R144] Moss E, Dubois-Comtois K, Tarabulsy C, St-Laurent D, Bernier A (2011). Efficacy of a home-visiting intervention aimed at improving maternal sensitivity, child attachment, and behavioral outcomes for maltreated children: A randomized control trial. Development and Psychopathology.

[R145] Neal TM, Slobogin C, Saks MJ, Faigman DL, Geisinger KF (2019). Psychological assessments in legal contexts: Are courts keeping “junk science” out of the courtroom?. Psychological Science in the Public Interest.

[R146] Newton RR, Litrownik AJ, Landsverk JA (2000). Children and youth in foster care: disentangling the relationship between problem behaviors and number of placements. Child Abuse & Neglect.

[R147] Nielsen L (2014). Woozles: Their role in custody law reform, parenting plans, and family court. Psychology, Public Policy, and Law.

[R148] Nicolini D, Mengis J, Swan J (2012). Understanding the role of objects in cross-disciplinary collaboration. Organization Science.

[R149] North G (2019). Assessing for bruises on the soul: identifying and evidencing childhood emotional abuse. Journal of Social Welfare and Family Law.

[R150] Oosterman M, Schuengel C, Slot NW, Bullens RA, Doreleijers TA (2007). Disruptions in foster care: A review and meta-analysis. Children and Youth Services Review.

[R151] Oosterman M, Schuengel C (2007). Autonomic reactivity of children to separation and reunion with foster parents. Journal of the American Academy of Child and Adolescent Psychiatry.

[R152] Owen MT, Cox MJ (1997). Marital conflict and the development of infant–parent attachment relationships. Journal of Family Psychology.

[R153] Padrón E, Carlson EA, Sroufe LA (2014). Frightened versus not frightened disorganized infant attachment: Newborn characteristics and maternal caregiving. American Journal of Orthopsychiatry.

[R154] Palacios J, Adroher S, Brodzinsky DM, Grotevant HD, Johnson DE, Juffer F, Martinez-Mora L, Muhamedrahimov RJ, Selwyn J, Simmonds J, Tarren-Sweeney M (2019). Adoption in the service of child protection: An international interdisciplinary perspective. Psychology, Public Policy, and Law.

[R155] Palacios J, Rolock N, Selwyn J, Barbosa-Ducharne M (2019). Adoption breakdown: Concept, research and implications. Research on Social Work Practice.

[R156] Pederson D, Moran G (1995). A categorical description of infant-mother relationships in the home and its relation to Q-sort measures of infant-mother interaction. Monographs of the Society for Research in Child Development.

[R157] Pederson DR, Moran G, Sitko C, Campbell K, Ghesquire K, Acton H (1990). Maternal sensitivity and the security of infant-mother attachment: A Q-sort study. Child Development.

[R158] Posada G, Lu T, Trumbell J, Kaloustian G, Trudel M, Plata S, Peña P, Perez J, Tereno S, Dugravier R, Coppola G (2013). Is the secure base phenomenon evident here, there, and anywhere? A cross-cultural study of child behavior and experts’ definitions. Child Development.

[R159] Posada G, Trumbell J, Noblega J, Plata S, Peña P, Carbonell OA (2016). Maternal sensitivity and child secure base use in early childhood: Studies in different cultural contexts. Child Development.

[R160] Pruett MK, Cowan CP, Cowan PA, Pradhan L, Robins S, Pruett KD, Drozd L, Saini M, Olesen N (2016). Parenting plan evaluations: Applied research for the family court.

[R161] Raub JM, Carson NJ, Cook BL, Wyshak G, Hauser BB (2013). Predictors of custody and visitation decisions by a family court clinic. Journal of the American Academy of Psychiatry and the Law Online.

[R162] Reijman S, Foster S, Duschinsky R (2018). The infant disorganised attachment classification: “Patterning within the disturbance of coherence”. Social Science & Medicine.

[R163] Robertson L, Broadhurst K (2019). Introducing social science evidence in family court decision-making and adjudication: Evidence from England and Wales. International Journal of Law, Policy and the Family.

[R164] Sagi A, van IJzendoorn MH, Aviezer O, Donnell F, Mayseless O (1994). Sleeping out of home in a kibbutz communal arrangement: It makes a difference for infant-mother attachment. Child Development.

[R165] Sallnäs M, Vinnerljung B, Kyhle Westermark K (2004). Breakdown of teenage placements in Swedish foster and residential care. Child & Family Social Work.

[R166] Salter EK (2012). Deciding for a child: a comprehensive analysis of the best interest standard. Theoretical Medicine and Bioethics.

[R167] Saunders R, Jacobvitz D, Zaccagnino M, Beverung L, Hazen N (2011). Pathways to earned-security: The role of alternative support figures. Attachment & Human Development.

[R168] Schneider CE (1991). Discretion, rules, and law: Child custody and the UMDA's best-interest standard. Michigan Law Review.

[R169] Schoemaker NK, Wentholt WG, Goemans A, Vermeer HJ, Juffer F, Alink LR (2019). A meta-analytic review of parenting interventions in foster care and adoption. Development and Psychopathology.

[R170] Schofield G, Walsh J (2010). Young carers – or children in need of care? Decision making for children of parents with mental health problems. Child & Family Law Quarterly.

[R171] Schore A, McIntosh J (2011). Family law and the neuroscience of attachment. Family Court Review.

[R172] Schuengel C, Bakermans-Kranenburg MJ, Van IJzendoorn MH (1999). Frightening maternal behavior linking unresolved loss and disorganized infant attachment. Journal of Consulting and Clinical Psychology.

[R173] Scott ES, Emery RE (2014). Gender politics and child custody: The puzzling persistence of the best-interest standard. Law & Contemporary Problems.

[R174] Scott S, Briskman J, Woolgar M, Humayun S, O’Connor TM (2011). Attachment in adolescence: overlap with parenting and unique prediction of behavioural adjustment. Journal of Child Psychology and Psychiatry.

[R175] Shemmings D (2018). Why social workers shouldn’t use ‘attachment’ in their records and reports. Community Care.

[R176] Shemmings D, Shemmings Y (2011). Understanding disorganized attachment: Theory and practice for working with children and adults.

[R177] Simpson J, Belsky J, Cassidy J, Shaver P (2016). Handbook of attachment: Theory, research, and clinical applications.

[R178] Skivenes M, Sørsdal LM, Falch-Eriksen A, Backe-Hansen E (2018). Human rights in child protection.

[R179] Smith G, Coffino B, Lieberman A, Van Horn P (2012a). Attachment and child custody: The importance of available parents. Parenting plan evaluations: Applied research for the family court.

[R180] Smith G, Caffino B, Van Horn P, Lieberman A, Kuehnle K, Drozd L (2012b). Parenting plan evaluations: Applied research for the family court.

[R181] Smyke AT, Zeanah CH, Gleason MM, Drury SS, Fox NA, Nelson CA, Guthrie D (2012). A randomized controlled trial comparing foster care and institutional care for children with signs of reactive attachment disorder. American Journal of Psychiatry.

[R182] Solomon J, Gunsberg L, Hymowitz P (2013). Handbook of divorce and custody.

[R183] Solomon J, Duschinsky R, Bakkum L, Schuengel C (2017). Toward an architecture of attachment disorganization: John Bowlby’s published and unpublished reflections. Clinical Child Psychology and Psychiatry.

[R184] Solomon J, George C (1999). The development of attachment in separated and divorced families: Effects of overnight visitation, parent, and couple variables. Attachment & Human Development.

[R185] Solomon J, George C, Solomon J, George C (2011). Disorganized attachment and caregiving.

[R186] Solomon J, George C, Cassidy J, Shaver PR (2016). Handbook of attachment: Theory, research, and clinical applications.

[R187] Spangler G, Fremmer-Bombik E, Grossmann K (1996). Social and individual determinants of infant attachment security and disorganization. Infant Mental Health Journal.

[R188] Spieker SJ, Crittenden PM (2018). Can attachment inform decision-making in child protection and forensic settings?. Infant Mental Health Journal.

[R189] Sroufe A, McIntosh J (2011). Divorce and attachment relationships: The longitudinal journey. Family Court Review.

[R190] Star SL (2010). This is not a boundary object: Reflections on the origin of a concept. Science, Technology, & Human Values.

[R191] Steele H, Murphy A, Bonuck K, Meissner P, Steele M (2019). Randomized control trial report on the effectiveness of Group Attachment-Based Intervention (GABI©): Improvements in the parent–child relationship not seen in the control group. Development and Psychopathology.

[R192] Steele H, Steele M (2017). Handbook of attachment-based interventions.

[R193] Steinbach A (2019). Children's and parents’ well-being in joint physical custody: A literature review. Family Process.

[R194] Stovall KC, Dozier M (2000). The development of attachment in new relationships: Single subject analyses for ten foster infants. Development and Psychopathology.

[R195] Stronach EP, Toth SL, Rogosch F, Cicchetti D (2013). Preventive interventions and sustained attachment security in maltreated children. D&P.

[R196] Swedish National Board of Health and Welfare (2018). Grundbok i BBIC – Barns Behov i Centrum.

[R197] Swedish National Board of Health and Welfare (2018). Metodstöd för BBIC.

[R198] Tan ES, McIntosh JE, Kothe EJ, Opie JE, Olsson CA (2018). Couple relationship quality and offspring attachment security: A systematic review with meta-analysis. Attachment & Human Development.

[R199] The National People’s Congress of the People’s Republic of China (2020). Chapter 4 Divorce, § 1084 & 1086. Civil Code of the People’s Republic of China.

[R200] The Supreme People’s Court of the People’s Republic of China (1993). A number of specific Opinions on the handling of child-rearing issues in divorce cases by the People’s Courts §4, Bulletin of the Supreme People’s Court of the People’s Republic of China.

[R201] Tornello SL, Emery R, Rowen J, Potter D, Ocker B, Xu Y (2013). Overnight custody arrangements, attachment, and adjustment among very young children. Journal of Marriage and Family.

[R202] Toussaint E, Florin A, Schneider B, Bacro F (2018). Les problèmes de comportement, les représentations d’attachement et le parcours de placement d’enfants relevant de la protection de l’enfance. Neuropsychiatrie de l’Enfance et de l’Adolescence.

[R203] Umemura T, Jacobvitz DB (2014). Nonmaternal care hours and temperament predict infants’ proximity-seeking behavior and attachment subgroups. Infant Behavior and Development.

[R204] Umemura T, Jacobvitz D, Messina S, Hazen N (2013). Do toddlers prefer the primary caregiver or the parent with whom they feel more secure? The role of toddler emotion. Infant Behavior and Development.

[R205] UN General Assembly (1989). Convention on the Rights of the Child.

[R206] USDHHS (2016). Determining the best interests of the child: State statutes.

[R207] Van der Asdonk S, de Haan WD, van Berkel SR, van IJzendoorn MH, Rippe RCA, Schuengel C, Kuiper C, Lindauer RJL, Overbeek M, Alink LRA Effectiveness of an attachment-based intervention for the assessment of parenting capacities in maltreating families: A randomized controlled trial. Infant Mental Health Journal.

[R208] Van den Dries L, Juffer F, Van IJzendoorn MH, Bakermans-Kranenburg MJ (2009). Fostering security? A meta-analysis of attachment in adopted children. Children and youth services review.

[R209] Van IJzendoorn MH (2005). Attachment in social networks: toward an evolutionary social network model. Human Development.

[R210] van IJzendoorn MH, Hein S, Weeland J (2019). New Directions for Child and Adolescent Development.

[R211] Van IJzendoorn MH, Bakermans-Kranenburg MJ, Zentner M, Shiner RL (2012). Handbook of temperament.

[R212] Van IJzendoorn MH, Bakermans-Kranenburg MJ, Duschinsky R, Goldman PS, Fox NA, Gunnar MR, Johnson DE, Nelson CA, Reijman S, Skinner GCM, Zeanah CH (2020). Institutionalisation and deinstitutionalisation of children I: A systematic and integrative review of evidence regarding effects on development. The Lancet Psychiatry.

[R213] Van IJzendoorn MH, Bakermans-Kranenburg MJ, Duschinsky R, Skinner GCM, Dwyer JG (2019). The Oxford handbook of children and the law.

[R214] Van IJzendoorn MH, Bakermans JJ, Steele M, Granqvist P (2018). Diagnostic use of Crittenden’s attachment measures in Family Court is not beyond a reasonable doubt. Infant Mental Health Journal.

[R215] Van IJzendoorn MH, Sagi A, Lambermon MWE, Pianta RC (1992). Beyond the parent: The role of other adults in children's lives New Directions for Child Development.

[R216] Van IJzendoorn MH, Schuengel C, Bakermans-Kranenburg MJ (1999). Disorganized attachment in early childhood: Meta-analysis of precursors, concomitants, and sequelae. Development and Psychopathology.

[R217] Van IJzendoorn MH, Steele M, Granqvist P (2018). On exactitude in science: A map of the empire the size of the empire. Infant Mental Health Journal.

[R218] Van IJzendoorn MH, Vereijken CM, Bakermans-Kranenburg MJ, Marianne Riksen-Walraven J (2004). Assessing attachment security with the attachment Q sort: Meta-analytic evidence for the validity of the observer AQS. Child Development.

[R219] Vaughn BE, Posada G, Verissimo M (2019). Secure base scripts and social competence in preschool children. Attachment & Human Development.

[R220] Warshak RA (2014). Social science and parenting plans for young children: A consensus report. Psychology, Public Policy, and Law.

[R221] Waters E, Deane KE (1985). Defining and assessing individual differences in attachment relationships: Q-methodology and the organization of behavior in infancy and early childhood. Monographs of the Society for Research in Child Development.

[R222] Waters E, Merrick S, Treboux D, Crowell J, Albersheim L (2000). Attachment security in infancy and early adulthood: A twenty-year longitudinal study. Child Development.

[R223] White S, Gibson M, Wastell D, Walsh P (2019). Reassessing attachment theory in child welfare.

[R224] Wilkins D (2012). Disorganized attachment indicates maltreatment: how is this link useful for child protection social workers?. Journal of Social Work Practice.

[R225] Wilkins D (2020). Disorganized attachment does not indicate child maltreatment. Journal of Social Work Practice.

[R226] Winnicott DW (1971). Playing and reality.

[R227] Woolgar M, Baldock E (2015). Attachment disorders versus more common problems in looked after and adopted children: Comparing community and expert assessments. Child and Adolescent Mental Health.

[R228] Wulczyn F, Kogan J, Harden BJ (2003). Placement stability and movement trajectories. Social Service Review.

[R229] Wulczyn F, Zimmerman E (2005). Sibling placements in longitudinal perspective. Children and Youth Services Review.

[R230] Zeanah CH, Chesher T, Boris NW, Walter HJ, Bukstein OG, Bellonci C, Benson S, Bussing R, Chrisman A, Hamilton J, Hayek M (2016). Practice parameter for the assessment and treatment of children and adolescents with reactive attachment disorder and disinhibited social engagement disorder. Journal of the American Academy of Child & Adolescent Psychiatry.

[R231] Zeanah CH, Larrieu JA, Heller SS, Valliere J, Zeanah CH (2000). Handbook of infant mental health.

[R232] Zeanah CH, Smyke AT, Koga SF, Carlson E, Bucharest Early Intervention Project Core Group (2005). Attachment in institutionalized and community children in Romania. Child Development.

[R233] Zimmermann P, Zimmermann P, Spangler G (2017). Feinfühlige Herausforderung: Bindung in Familie, Kita, Kinderheim und Jugendhilfe.

